# ADMET evaluation in drug discovery. 20. Prediction of breast cancer resistance protein inhibition through machine learning

**DOI:** 10.1186/s13321-020-00421-y

**Published:** 2020-03-05

**Authors:** Dejun Jiang, Tailong Lei, Zhe Wang, Chao Shen, Dongsheng Cao, Tingjun Hou

**Affiliations:** 1 Hangzhou Institute of Innovative Medicine, College of Pharmaceutical Sciences, Zhejiang University, Hangzhou, 310058 Zhejiang People’s Republic of China; 2 Xiangya School of Pharmaceutical Sciences, Central South University, Changsha, 410004 Hunan People’s Republic of China

**Keywords:** Breast cancer resistance protein, Multi-drug resistance, Machine learning, Extreme gradient boosting, Ensemble learning, ADMET, Deep learning

## Abstract

Breast cancer resistance protein (BCRP/ABCG2), an ATP-binding cassette (ABC) efflux transporter, plays a critical role in multi-drug resistance (MDR) to anti-cancer drugs and drug–drug interactions. The prediction of BCRP inhibition can facilitate evaluating potential drug resistance and drug–drug interactions in early stage of drug discovery. Here we reported a structurally diverse dataset consisting of 1098 BCRP inhibitors and 1701 non-inhibitors. Analysis of various physicochemical properties illustrates that BCRP inhibitors are more hydrophobic and aromatic than non-inhibitors. We then developed a series of quantitative structure–activity relationship (QSAR) models to discriminate between BCRP inhibitors and non-inhibitors. The optimal feature subset was determined by a wrapper feature selection method named rfSA (simulated annealing algorithm coupled with random forest), and the classification models were established by using seven machine learning approaches based on the optimal feature subset, including a deep learning method, two ensemble learning methods, and four classical machine learning methods. The statistical results demonstrated that three methods, including support vector machine (SVM), deep neural networks (DNN) and extreme gradient boosting (XGBoost), outperformed the others, and the SVM classifier yielded the best predictions (MCC = 0.812 and AUC = 0.958 for the test set). Then, a perturbation-based model-agnostic method was used to interpret our models and analyze the representative features for different models. The application domain analysis demonstrated the prediction reliability of our models. Moreover, the important structural fragments related to BCRP inhibition were identified by the information gain (IG) method along with the frequency analysis. In conclusion, we believe that the classification models developed in this study can be regarded as simple and accurate tools to distinguish BCRP inhibitors from non-inhibitors in drug design and discovery pipelines.
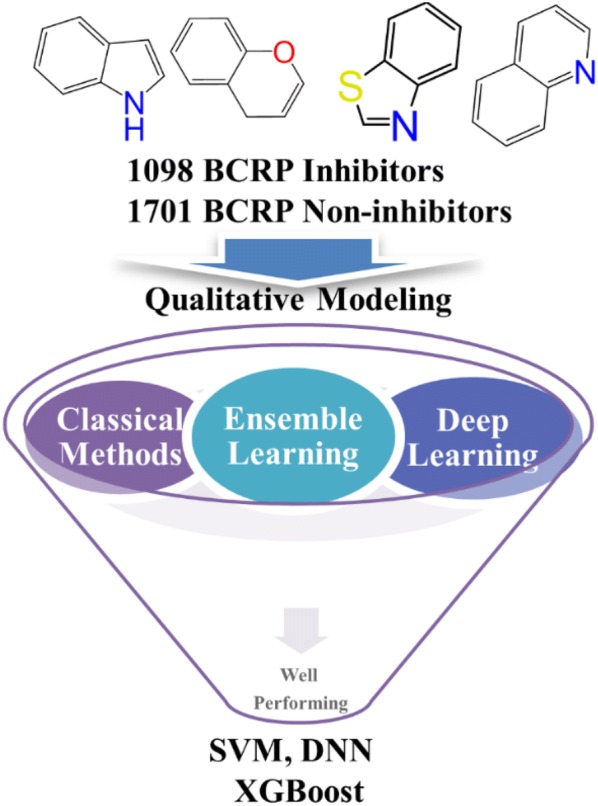

## Introduction

The breast cancer resistance protein (BCRP/ABCG2) is the second member of the G subfamily of the ATP-binding cassette (ABC) transporter superfamily. It is widely distributed and expressed in many normal tissues, especially the small intestine, liver, brain endothelium, and placenta [1]. BCRP functions as a xenobiotic efflux pump by exporting antineoplastic drugs out of cells, such as topotecan and mitoxantrone, and its overexpression or activation results in multi-drug resistance (MDR) to many chemotherapeutic agents [2–4]. It is also a vital constituent part of blood–tissue barriers and is closely involved in absorption blocking and excretion enhancing. Therefore, BCRP has been recognized as an important player in drug absorption, distribution, metabolism, excretion, and toxicity (ADMET) [5, 6], and has also been regarded by the Food and Drug Administration (FDA) as one of the key drug transporters in clinical drug disposition [4, 7].

Many drugs, such as mitoxantrone and topotecan, have been identified as BCRP substrates [3, 8], and BCRP inhibitors can enhance the bioavailability of these drugs [9, 10]. For instance, the co-administration of topotecan (an anti-cancer drug) and GF120918 (a BCRP inhibitor) resulted in a significant increase of the systemic exposure of topotecan [9]. Gefitinib, a potent BCRP inhibitor, could considerably improve the bioavailability of irinotecan in mice [10]. Therefore, the discovery of novel BCRP inhibitors can contribute to the design of better therapeutic strategies for cancer treatment and identification of potential drug–drug interactions [11]. Many experimental techniques have been employed to measure the BCRP inhibition of drugs or drug candidates, but these assays are expensive, resource-intensive, and time-consuming. Thus, it is of critical importance to develop in silico models to predict BCRP inhibition in the process of drug design and discovery in order to reduce the failure rates and avoid adverse drug–drug interactions.

Quantitative structure–activity relationship (QSAR) analysis is one of the most classical techniques in the realm of computer-aided drug design (CADD) to correlate the chemical structures of molecules with their bioactivities. To date, numerous computational QSAR models based on various machine learning (ML) approaches and pharmacophore modeling have been proposed to predict BCRP inhibition [12–22] For example, in 2005, based on a dataset of 25 flavonoids, Zhang et al. [15] built a multiple linear regression model to quantitatively predict the BCRP inhibition of flavonoids. In 2007, Matsson and co-workers used the orthogonal partial least-squares projection to latent structures discriminant analysis (OPLS-DA) to construct a classification model to differentiate BCRP inhibitors from non-inhibitors with predictive accuracies of 93% and 79% for the training and test sets, respectively [16]. It is surprising that an easily interpretable model could be established based on only two descriptors: octanol–water partition coefficient at pH 7.4 and molecular polarizability. In 2013, Pan et al. generated the Bayesian classification and pharmacophore models based on a training set of 124 BCRP inhibitors and non-inhibitors, and the best Bayesian classification model achieved a Matthews correlation coefficient (MCC) of 0.69 for the test set with 79 compounds and the best pharmacophore model yielded a MCC of 0.29 for the same test set [11]. In 2014, based on a large dataset containing 780 diverse compounds, Montanari et al. also used the naïve Bayesian approach to establish a model with an encouraging global accuracy of 0.92 but a relatively low AUC (the area under the receiver operating characteristic curve) value of 0.854 for the training set under tenfold cross-validation. In addition, this model was not validated by a test set [20]. In 2015, based on a training set of 197 compounds, Belekar et al. developed a number of models to distinguish BCRP inhibitors from non-inhibitors by using support vector machine (SVM), k-nearest neighbor (k-NN), and artificial neural networks (ANN), and the models achieved global accuracies of 0.828–0.878 for the test set with 99 compounds and 0.745–0.775 for the validation set with 99 compounds [21]. Recently, Montanari et al. developed a computational model to qualitatively predict BCRP inhibition by using logistic regression (LR) based on a relatively large dataset with 978 compounds [22], and the tenfold cross-validation of the LR model for the training set yielded a MCC of 0.65 and an AUC of 0.90. Apparently, most reported computational models for differentiating BCRP inhibitors from non-inhibitors were developed based on relatively small datasets (less than 1000 compounds) or congeneric compounds and suffered from relatively low accuracy. As a result, the reliability and general applicability of the developed models may be limited. Therefore, it is an urgent need to develop computational models with high reliability and robustness to predict BCRP inhibition based on an extensive dataset.

Recently, new artificial intelligence (AI) methods, such as ensemble learning represented by extreme gradient boosting (XGBoost) and deep learning (DL) represented by deep neural networks (DNN), have been widely employed in CADD, such as drug-target interactions prediction [23], cancer diagnosis [24], ADMET predictions and QSAR modeling [25–33]. DL is a categorization of artificial neural networks-based (ANN-based) ML methods that gradually extract features from the raw input using multiple layers. The application of DL in drug discovery was hindered by computability, but it can be well alleviated by using graphics processing units (GPUs) acceleration [34]. Ensemble learning represented by XGBoost attracted the attention of researchers in drug discovery due to its accurate predictions and low computational cost, and some studies reported that XGBoost could achieve comparative or even better performance than other widely-used ML methods such as RF and DNN [31]. Thus, it is a recognized need to explore how well those state-of-the-art methods perform in the prediction of BCRP inhibition. In this study, we reported a large and structurally diverse dataset with 1098 BCRP inhibitors and 1701 non-inhibitors. Then, based on the extensive dataset, the classification models to discriminate between BCRP inhibitors and non-inhibitors were developed by using seven ML approaches, including a representative DL method: DNN, two representative ensemble learning methods: stochastic gradient boosting (SGB) and XGBoost, and four traditional ML methods: naive Bayes (NB), k-NN, regularized logistic regression (RLR), and SVM. The optimal feature subset used in model development was determined by a wrapper feature selection method that couples simulated annealing (SA) algorithm and random forest (RF) and the hyper-parameters were determined by the Bayesian optimization technique. To our knowledge, there are relatively few studies devoted to using new AI methods, such as ensemble learning and deep learning, to the prediction of BCRP inhibition. The robustness and reliability of the models were validated internally and externally, and the statistical results demonstrated that one traditional ML method (SVM) and two new ML methods (DNN and XGBoost) could generate reliable classification models for the prediction of BCRP inhibition, and the SVM model achieved the best predictions. The identification and analyses of representative features for different models were accomplished through a perturbation-based method. Furthermore, the important structural fragments related to BCRP inhibition were recognized by the information gain method along with the frequency analysis, which may provide valuable clues for the design of potent BCRP inhibitors and avoiding undesirable drug–drug interactions.

## Materials and methods

### Dataset preparation

In this study, three data sources were integrated to build up an extensive BCRP inhibition dataset. The first data source is the dataset of 978 compounds reported by Montanari et al. [20], and it contains 433 inhibitors and 545 non-inhibitors. The second data source that has 765 BCRP inhibitors and 220 non-inhibitors were manually collected from 51 literatures [22, 35–84]. However, considering the fact that BCRP non-inhibitors should be much more than inhibitors, the third data source containing 1000 non-inhibitors was also integrated into the final dataset. The 1000 non-inhibitors were extracted from the PubChem database (PubChem AID: 1325), and they have the lowest efflux pump activity, which was defined as “% inhibition of efflux pump activity”. According to the authors’ statement, compounds with percentage inhibition lower than 80% were decisively inactive. The molecules from the above three data sources were integrated into a single dataset and the duplicated molecules with incoherent labels were removed. Then, the salts, mixtures, and metal–organic compounds were eliminated using the wash tool in the Molecular Operating Environment (MOE) software package (version 2015), and then all the compounds were minimized with the MMFF94 force field [85]. The final dataset contains 1098 BCRP inhibitors and 1701 BCRP non-inhibitors. The whole dataset can be accessed from Additional file 1: Table S1 or the supporting website: http://cadd.zju.edu.cn/pkkb/. As far as we know, the dataset reported in this study is the largest one of its kind available to the public. The BCRP inhibitors were labeled as 1 and non-inhibitors were labeled as 0.

### Generation of molecular descriptors and fingerprints

A total of 379 molecular descriptors, which characterize the physicochemical, structural and drug-like properties of the studied compounds, were calculated using the MOE 2015 as the molecular features. According to many previous studies, the addition of molecular fingerprints can improve the performance of QSAR modeling [86–88]. Therefore, the PubChem fingerprints (PubchemFP) with 881 substructures generated by PaDEL-Descriptor [89], which characterize 2-dimensional substructures in a binary format, were also used as molecular features. The molecular features with missing values were removed. Then, the features that have one unique value (i.e., zero-variance features) or features have very few unique values relative to the number of compounds or features have a large frequency gap between the most common value and the second most common value were removed by the *nearZeroVar* function in the *caret* package of R (version 3.5.3 ×64). In addition, the correlation between any two features was calculated and the feature that has high correlation (*r* > 0.90) with another feature was removed. After this step, 382 molecular features were retained and the whole dataset was randomly divided into the training and test sets with a ratio of 4:1 using a stratified sampling method. As a result, the training set contains 2240 compounds (879 inhibitors and 1361 non-inhibitors), and the test set contains 559 compounds (219 inhibitors and 340 non-inhibitors).

### Feature selection by rfSA

All the calculated molecular features cannot be used for the QSAR modeling due to the curse of dimension for high-dimensional data and the phenomenon of over-fitting. Therefore, a wrapper feature selection method named rfSA (SA algorithm coupled with RF) was used to remove redundant or irrelevant features without sacrificing too much information based on the training set. The SA algorithm is used to search the feature subset from the origin feature space and the performance of the RF model developed from the selected feature subset is used to guide the search. As a common method for combinatorial optimization problems, SA has good capability to avoid being trapped into local minima [90]. In the feature subset search process, the RF model developed from a new feature subset selected by the SA algorithm is compared with that developed from the previous feature subset identified in the previous step according to the RF model performance (e.g. accuracy of RF model). If the new feature subset performs better, it would be accepted, otherwise acceptance probability is calculated based on the accuracy difference between the two RF models developed from the two feature subsets and the current iteration of the search. This process is repeated until the stopping criteria are satisfied. The rfSA was implemented using a built-in *safs* function in the *caret* package of R (version 3.5.3 ×64). Here, the resample method was set as fivefold cross-validation with five repetitions to guarantee the statistical significance, where four-fifth of the training set (internal set) was used in the feature subset search conducted by SA and the remaining one-fifth (external set) was used to estimate the external accuracy. The best iteration of SA was determined by maximizing the external accuracy. The maximum iterations of the SA optimization were set to 1000. More descriptions about the feature selection process can be found in the documentations [91, 92].

### QSAR model construction and hyper-parameters optimization

**Fig. 1:**
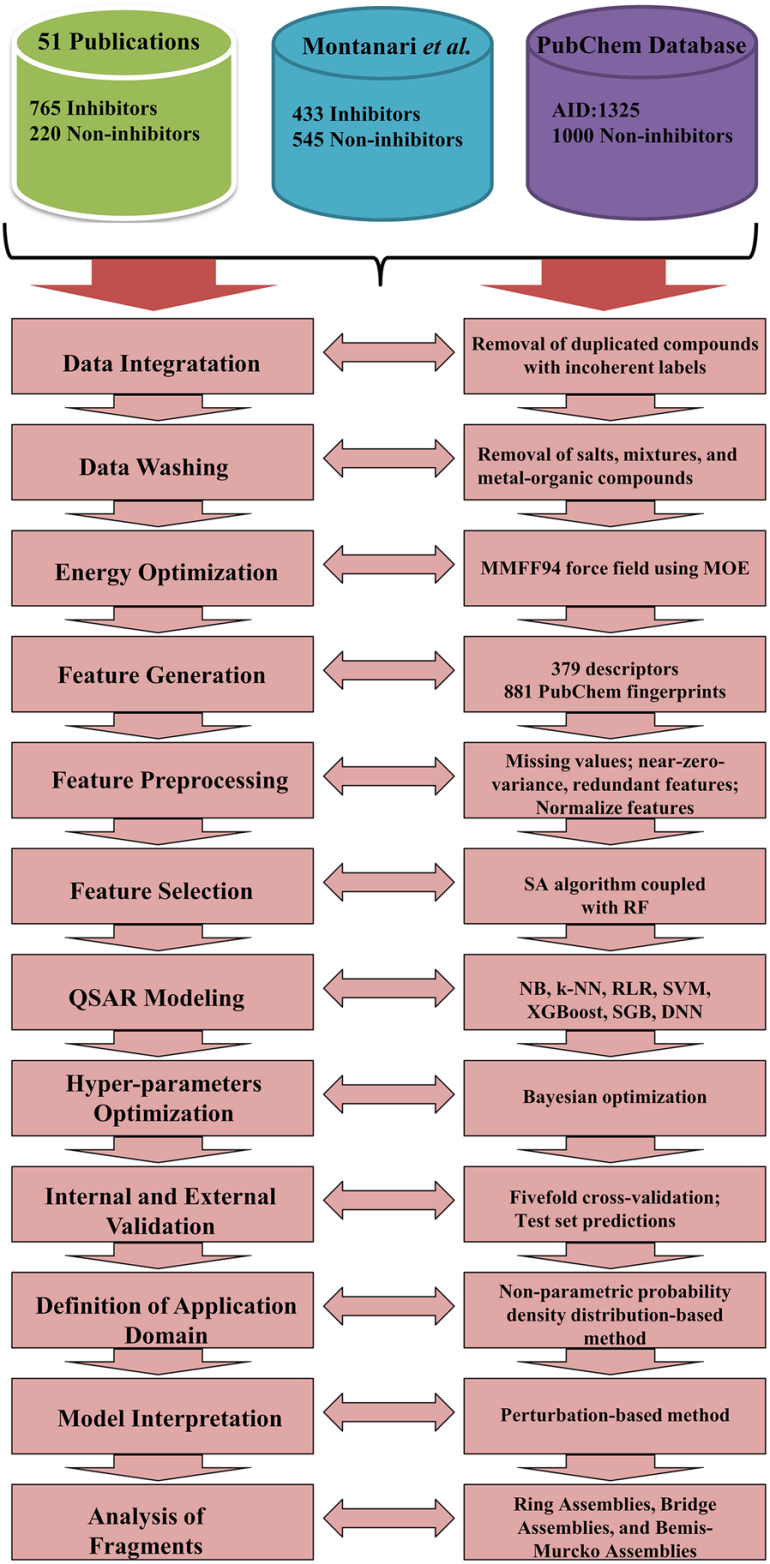
The workflow of QSAR modeling

Here, seven ML methods were employed to develop the classification models to discriminate BCRP inhibitors and non-inhibitors, including a representative DL method (DNN), two representative ensemble learning methods (SGB and XGBoost), and four traditional ML methods (NB, k-NN, RLR and SVM). The DNN method was implemented in the *h2o* package of R (version 3.5.3 ×64), and the other six ML methods were implemented in the *caret* package of R (version 3.5.3 ×64). The *caret* package provides miscellaneous functions for building classification and regression models and focuses on simplifying model training at the same time. The whole QSAR modeling pipeline is presented in Fig. 1. The source code that implements the workflow is available in the supplementary information (Additional file 2).

#### Naive Bayes (NB)

The NB algorithm is a simple and interpretable probabilistic classification method, and it estimates the corresponding class probability for an instance represented by conditionally independent feature variables based on the Bayes’ theorem. Despite the simple theorem and oversimplified assumptions, NB has been extensively used in classification and achieved outstanding performance in many intricate real-world situations, such as text classification. In addition, NB is fast and efficient for large datasets, and it is less affected by “curse of dimensionality” when a large number of descriptors are used [93]. The detailed descriptions of the NB algorithm were documented previously [88].

#### k-Nearest neighbors (k-NN)

The k-NN algorithm is a commonly used non-parametric supervised learning approach for classification and regression [94]. The principle of this algorithm is to find the *k* closest training instances when a test instance is given and this test instance is predicted based on the information of the closest training instances. In our study, the “weighted voting method” was employed, which weights the contributions of the *k* closest instances using a distance weighting function, where the closest instance contributes most to the voting and the furthest instance contributes least.

#### Regularized logistic regression (RLR)

As an efficient and simple classification methods, the logistic regression (LR) algorithm uses the logistic function as the link function of generalized linear model [22, 95]. It is suited for conducting regression analysis where the response variable is binary. Different from conventional linear regression which fits a straight line or hyperplane, LR converts the output of a linear regression model to values between 0 and 1 using a logistic function. In this study, a regularization term by penalizing high values of the optimized parameters was introduced into the cost function of the LR algorithm to prevent over-fitting, which is the so-called regularized logistic regression (RLR).

#### Support vector machine (SVM)

Support vector machine is one of the most popular ML approaches in QSAR modeling. It is appropriate to develop the classification models for complex but small- or medium-sized datasets. The fundamental principle of SVM is to find a separating hyper-plane with maximum margin in the feature space for classifying instances of different categories, and the classification results produced by this hyper-plane should be insusceptible to local perturbations of training instances. In this study, the radial basis function (RBF) was used as the kernel.

#### Ensemble learning

Ensemble learning is a group of popular algorithms that can produce a strong learner in the form of an ensemble of weak learners. Gradient boosting is one of the most representative ensemble learning methods and it improves the strong learner gradually through fitting the new weak learner to residuals: an approximation of the negative gradient of the loss function being minimized. Here two gradient boosting-based methods, SGB and XGBoost, were employed to build the classification models. In SGB, a subsample of the training data is selected randomly to fit the new weak learner and update the strong learner for the current iteration, and this randomized approach increases both the approximation accuracy and execution speed of gradient boosting [96]. In XGBoost, some optimization algorithms are introduced based on the gradient boosting framework, such as minor improvements in the loss function by penalizing the complexity of the model, introduction of shrinkage and column subsampling for further preventing over-fitting, and employment of sparsity-aware split finding technique for efficient training on sparse data, etc. The significant predictive power of XGBoost on QSAR modeling has been illustrated in previous studies [29, 31].

#### Deep neural networks (DNN)

Deep learning has revolutionized many applications such as natural language processing, computer vision, and speech recognition due to its capability to learn complicated and rapidly-varying non-linear functions and extract a hierarchy of useful features from input [97]. DNN is one of the typical DL models which have an input layer, an output layer, and many hidden layers (typically more than three hidden layers). It is inspired from biological neurons networks and the basic component in DNN is the neuron model. In 2012, Merck sponsored a molecular activity challenge at the Kaggle data mining platform, and the competition was won by a group that utilized DNN as the main ML technique [25]. In addition, the Tox21 data challenge 2014 for toxicity assessment was won by a group that also utilized DNN as the main ML technique [26]. In DNN, each neuron receives the input signals from its connected neurons, and those input signals are multiplied by their respective weights and then summed up. The weighted sum plus the bias in each neuron to feed into the activation function and produce an output. Here the weights and biases in each neuron are the parameters in DNN and they are typically learned by error back-propagation algorithm in combination with optimization method such as stochastic gradient descent. It is commonly known that DNN learns to pick up important features from the original feature space by down-weighing irrelevant features. However, for the sake of fairness, DNN was trained based on the selected features from the training set although it seems redundant or not necessary.

#### Model hyper-parameters optimization

The performance of the models developed by ML approaches are closely related to important hyper-parameters. In this study, the hyper-parameters were optimized by the Bayesian optimization (aka model-based optimization) algorithm based on the MCC value of the fivefold cross-validation to the training set [98]. As a compelling global optimization method for black-box functions, the Bayesian optimization framework can obtain an ideal solution only after a few objective function evaluations by designing appropriate surrogate model and acquisition function. Before Bayesian optimization, a coarse hyper-parameter tuning based on grid search within relatively wide ranges was performed to determine a smaller region of hyper-parameters where the models work well. The Bayesian optimization was then used to zoom into these smaller regions and find more optimal settings of hyper-parameters. The Bayesian optimization algorithm was implemented by the *mlrMBO* package in R (version 3.5.3 ×64). The search space of the Bayesian optimization and the optimal hyper-parameters for all the studied models are summarized in Additional file 1: Table S2. It should be noted that some other important hyper-parameters of the DNN model were fixed in our study. Here, the optimizer of the DNN modeling was set to an adaptive learning rate algorithm: *ADADELTA*, and the activation function was set to *ReLU* which is highly recommended for DNN according to the previous studies [25, 99]. All the tested DNN configurations were trained for 300 epochs.

### Statistical validation of the QSAR models

According to the Organization for Economic Co-operation and Development (OECD) Validation Principle [4, 100], a QSAR model should be associated with appropriate measures of goodness-of-fit, robustness, and predictivity. Here, the random fivefold cross-validation of the training set was used to evaluate the robustness of each model, and the external validation given by the predictions to the test set was used to measure the actual predictivity of each model. In addition to the random fivefold cross-validation, the performance of the seven studied ML algorithms was also evaluated by the cluster cross-validation method proposed by Mayr et al. [101, 102]. The agglomerative hierarchical clustering using complete linkage was used to identify different compound clusters. The distance between any two compounds was measured by the Tanimoto similarity based on the PubChem fingerprints. The maximum distance between any identified clusters was set to 0.7 and the generated smaller compound clusters were distributed across the fivefolds randomly. The following statistical parameters based on the confusion matrix were used to assess the performance of each model: confusion matrix [true positives (TP), true negatives (TN), false positives (FP), false negatives (FN)], global accuracy (GA), balanced accuracy (BA), Matthews correlation coefficient (MCC), and the area under the receiver operating characteristic (ROC) curve (AUC). The GA, BA and MCC are defined as following:

1 $$GA = \frac{TP + TN}{TP + TN + FP + FN}$$

2 $$BA = 0.5 \times \left( {\frac{TP}{TP + FN} + \frac{TN}{TN + FP}} \right)$$

3 $$MCC = \frac{TP \times TN - FN \times FP}{{\sqrt {\left( {TP + FN} \right)\left( {TP + FP} \right)\left( {FN + TN} \right)\left( {TN + FP} \right)} }}.$$

Moreover, the residual (the binary cross entropy was used as the residual function in this study) distribution plots for the fivefold cross-validated training set and the test set were used to further diagnose the quality of the classification models. Different from the confusion matrix based statistical parameters which only supports the predicted class of a compound, it is capable of providing information about the predicted class probability distribution of a compound. For example, for an inhibitor: I [the class probability distribution is (0,1)] and a non-inhibitor: NI [the class probability distribution is (1,0)], the predictions (class probability) for I and NI given by model A are (0.4, 0.6) and (0.65, 0.35), respectively and those given by model B are (0.1, 0.9) and (0.95, 0.05), respectively. Both models give the same confusion matrix and the statistical parameters would be identical [for the majority of ML approaches, the class of a certain compound was derived from its class probability distribution. If the probability for a certain class is larger than or equal to a certain threshold (generally 0.5), this compound would be predicted as the corresponding class], which model should be used in this case? Actually, we still believe that model B is better than model A because the residual to the referenced class probability distribution of model B is smaller than that of model A. In this study, the binary cross entropy was used as the residual function, which was defined as:

4 $$CE_{binary} = - \left[ {y \times \log \left( p \right) + \left( {1 - y} \right) \times \log \left( {1 - p} \right)} \right]$$

where *y* is the true class label of compound (1 for inhibitor and 0 for non-inhibitor); *p* is the probability of being inhibitor for a compound and the greater the binary cross entropy is, the larger the residual is, and vice versa.

### Definition of application domain (AD)

Defining AD is an important step for QSAR modeling in the OECD guidelines because a developed model can only be expected to give reliable predictions for query chemicals that fall within the AD [100]. In this study, the non-parametric probability density distribution-based method was applied to identify the AD of a QSAR model. This method estimates the probability density function for the given data and it can be mainly classified into two categories: parametric and non-parametric approaches. Parametric approaches assume the data distribution follows a standard distribution, such as Gaussian or Poisson distribution. Non-parametric approaches do not make any assumptions about the data distribution and they can estimate the probability density solely from data [103, 104]. An advantage of non-parametric approaches is that they can identify internal empty spaces, and it has been argued that they are more accurate and appropriate than other common approaches, such as the range, distance and leverage approaches [103]. More details of the probability density distribution-based method have been documented in literature [103, 104]. The AD analysis was conducted by the AMBIT Discovery software (version 0.04) [105].

### Interpretation of QSAR models

Identification and analysis of important features may contribute to a better understanding of QSAR models and provide new domain knowledge. Here the agnostic feature importance assessment algorithm, a perturbation-based method, was employed to identify the most important features for different well-performing QSAR models. This algorithm is model agnostic and not relevant to the specific structure of a model. A couple of previous studies only identified and analyzed the important features given by feature selection procedure for QSAR models [21, 29, 106] but did not explore the relationships between features and different QSAR models. It is quite possible that a set of important descriptors/fingerprints for a well-performing QSAR model may be not important to another QSAR model. In view of that, this perturbation-based model-agnostic method was used to recognize important features for different well-performing QSAR models and further compare the important features between models for interpreting how different QSAR models handle particular features. The basic principle of this method is to evaluate how much the model performance is gone from the performance of the original model after the selected feature is perturbed or resampled. The more the model performance is gone, the more important the feature is, and vice versa. In a more specific way, $L = L\left( {Y,f\left( X \right)} \right)$ denotes the loss function of a model where $Y$ is the observations for all instances and $f\left( X \right)$ is the predictions of all instances. The flowing steps were conducted for each single feature $v_{i}$ in the $V$: first, a perturbed data $X^{*, - i}$ with feature $v_{i}$ perturbed or resampled was generated; second, the model predictions $f\left( {X^{*, - i} } \right)$ on the perturbed data were calculated; then, the loss function $L^{*, - i} = L\left( {Y,f\left( {X^{*, - i} } \right)} \right)$ on the perturbed data was obtained; finally, the feature importance of $v_{i}$ is measured as the difference or ratio of the original loss $L$ and loss on perturbed data $L^{*, - i}$ [107, 108]. Here, the binary cross entropy was used as the loss function, and the identification and analysis procedure was implemented in the *DALEX* package of R (version 3.5.3 ×64). In addition, the analysis for each single feature was repeated 10 times to guarantee the repeatability of results due to the perturbation randomness and the importance score was obtained by averaging the results of the 10 times repetitions.

### Analysis of important fragments to characterize BCRP inhibitors and non-inhibitors

In order to better understand important chemical features related to BCRP inhibition, each compound was decomposed into three structural fragments and analyzed. The generated structural fragments include contiguous ring systems (Ring Assemblies), contiguous ring systems that share two or more bonds (Bridge Assemblies), and contiguous ring systems plus chains that link two or more rings (BemisMurcko Assemblies). The details of these three structural fragments can be found in previous studies [109]. The structural fragments for all the compounds were generated using the *Generate Fragments* component in Pipeline Pilot 2017, and the *Merge Molecules* component was then used to merge records containing identical fragments into a single record for the next statistical analysis.

The important fragments that have large contributions to BCRP inhibitors or non-inhibitors were identified by the information gain (IG) method coupled with frequency analysis [110, 111]. The IG value of a fragment was used to measure its importance to the classification system, and the fragments with low IG values are less effective in a classification system and vice versa. In this study, the IG value of a fragment was calculated as followings:

5 $${\text{IG}}\left( {\text{fragment}} \right) = Ent\left( D \right) - \mathop \sum \limits_{v = 0}^{V} \left( {\frac{{N^{v} }}{N} \times Ent\left( {D^{v} } \right)} \right)$$

6 $$Ent\left( D \right) = - \mathop \sum \limits_{k = 0}^{K} p_{k} \times \log_{2} p_{k}$$

where $V$ represents the possible value of a fragment (0 or 1); 0 represents the fragment is absent in a compound and 1 represents the fragment is present in a compound. $N$ denotes the total number of compounds; $N^{v}$ is the number of compounds where the fragment is present or absent. $Ent\left( D \right)$ and $Ent\left( {D^{v} } \right)$ represent the information entropy and conditional entropy of all the compounds, respectively, and both of them were calculated by Eq. 6, where $K$ represents the classes of the compounds (1 represents inhibitors and 0 represents non-inhibitors), and $p_{k}$ is the ratio of each class compounds. The frequency of a fragment was defined as:

7 $${\text{frequency of a fragment }} = \frac{{N_{fragment\_class} \times N_{total} }}{{N_{fragment\_total} \times N_{class} }}$$

where $N_{fragment\_class}$ is the number of compounds containing the fragment in each class; $N_{total}$ is the total number of compounds; $N_{fragment\_total}$ is the total number of compounds containing the fragment; $N_{class}$ is the number of compounds in each class. If the occurrence of a fragment is more frequent in inhibitors than in non-inhibitors, this fragment was considered as an important fragment for the inhibitors and vice versa.

## Results and discussion

### Dataset collection

Except 978 BCRP inhibitors and non-inhibitors reported by Montanari et al. [20] and 1000 non-inhibitors extracted from the PubChem database (PubChem AID: 1325), 985 BCRP inhibitors and non-inhibitors collected manually from 51 publications were integrated into the whole BCRP inhibition dataset. The basic information of the compounds in these 51 publications, including compound name, compound structure, activity source, activity index, and reported value, were collected manually and stored into a Molecular Operating Environment (MOE) database. The reported inhibition activity index is represented by IC_50_, EC_50_, %inhibition at a given concentration, or substrate fold-increase, etc. The experimental assays to determine the BCRP inhibition activity can be roughly categorized into two types: (1) fluorescent or radioactivity substrate intracellular accumulation assay and (2) membrane vesicular transport assay. In the fluorescent or radioactivity substrate intracellular accumulation assay, cells overexpressing BCRP are incubated with both fluorescent or radiolabeled substrate (pheophorbide A, mitoxantrone, BODIPY-prazosin, or Hoechst 33342) and the tested compound [52], and the quantity of fluorescence or radioactivity accumulation was measured and it is related to the inhibitory effect of the tested compound. The membrane vesicular transport assay employs inside-out-oriented membrane vesicles, prepared from cells expressing BCRP, to measure the influx of radioactive substrate such as ^3^H-methotrexate [19], and it can be used as an indirect (inhibition-type) way to determine the inhibitory effect of a tested compound [112].

Because the inhomogeneity of the experimental data, we used the following rules to convert the reported values into the inhibitor and non-inhibitor classes. First, the suggestions recommended by the authors were used to assign the possible classification threshold or the compound category. It should be noted that the suggestions recommended by the authors may be arbitrary, but it is the situation we are facing now. For example, Gros et al. [40] checked if some compounds could inhibit the transport activity of BCRP, and the authors found that a portion of the studied compounds had no any effect on the mitoxantrone efflux and therefore they were not inhibitors of BCRP. Naturally, those compounds were considered to be non-inhibitors in our study. Elsby et al. [36] suggested a rheumatoid arthritis candidate drug (AZD9056) was an inhibitor of BCRP based on biological activity assay. Similarly, AZD9056 was considered as an inhibitor. The study reported by Sjostedt et al. [73] defined compounds that result in normalized transport values below 50% as BCRP inhibitors. Second, the IC_50_ or EC_50_ value was used to categorize a compound. More specifically, a compound with IC_50_ or EC_50_ < 10 μM was classified as an inhibitor, and a compound with IC_50_ or EC_50_ > 50 μM was classified as a non-inhibitor, and those compounds with IC_50_ or EC_50_ between 10 and 50 μM were excluded because the testing results are susceptible to experimental conditions. In addition, for compounds that have multiple reported IC_50_ or EC_50_ values, the average values were used to assign inhibitors or non-inhibitors. But if the discrepancy between multiple values was significant for a compound, such compound was excluded. Third, for the activity index: %inhibition at a given concentration, only the concentration at l0 μM was taken into consideration. A compound with %inhibition at 10 μM > 50% was classified as an inhibitor, and the compound with %inhibition at 10 μM < 25% was classified as a non-inhibitor. Similarly, the compounds with %inhibition at 10 μM ranging from 25 to 50% were removed from the dataset owing to their susceptibilities to experimental conditions.

Unfortunately, some compounds still could not be categorized after this step. For those compounds, the “outcome” column of the PubChem database was employed to define the compound category. The “outcome” column can be one of the following four values, including “Active”, “Inactive”, “Inconclusive”, and “Unspecified”, and it is in some ways equivalent to the author’s subjective definition of what the compound is. In the light of the “outcome” column, the compounds with the “Active” label were considered as inhibitors in our study, and those “Inactive” labeled compounds were considered as non-inhibitors. Certainly, “Inconclusive” or “Unspecified” labeled compounds were removed.

It should be noted that the inhibitory mechanisms of different compounds towards BCRP may vary from one to another and the BCRP inhibitors can be approximately classified into the following three categories: [4] (a) compounds are regarded as “general” inhibitors to BCRP if they are able to inhibit ATPase activity of BCRP, such as the compounds No. 87 (fumitremorgin C) and No. 25 (Ko143) in the final dataset; (b) compounds were considered as competitive inhibitors if they are the substrates of BCRP and capable of competitively binding to the substrate site to block the transport of substrates, such as the compounds No. 1539 (dipyridamole), No. 440 (2-(2-methoxyphenyl)-*N*-(3-nitrophenyl)quinazolin-4-amine), and No. 259 (2-(3,4-dimethoxyphenyl)-*N*-(4-nitrophenyl)quinazolin-4-amine) in the final dataset; (c) compounds are not the substrates of BCRP, but they can bind to BCRP and induce conformational changes to affect the transport function of BCRP, for example, the compound No. 1633 (nelfinavir) in the final dataset. To better understand the complicated molecular mechanisms of BCRP inhibition, it is highly valuable to develop computational classification model for “general” inhibitors and “non-general” inhibitors, respectively. However, considering the fact that the inhibitory mechanisms for most inhibitors in the final dataset are still not clear, in this study, “global” classification models were developed to discriminate BCRP inhibitors and BCRP non-inhibitors with no consideration of their specific inhibitory mechanisms.

**Fig. 2:**
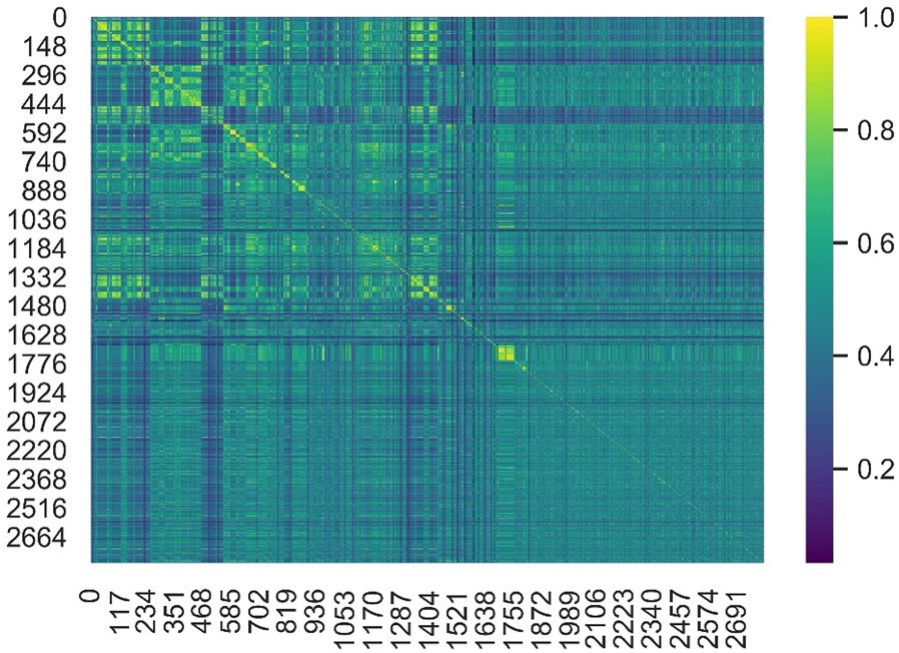
The chemical diversity of the final dataset evaluated by the Tanimoto similarities between any two of the compounds. The Tanimoto index was calculated based on the 881 PubChem fingerprints

The Tanimoto similarity index based on the 881 PubChem fingerprints was used to evaluate the chemical diversity of the final dataset. As shown in Fig. 2, for the overwhelming majority of the compounds, the Tanimoto similarities between any two of compounds are relatively low and the average Tanimoto similarity for the whole dataset is 0.464, highlighting the structural diversity of the final dataset.

### The impact of simple molecular descriptors on BCRP inhibition

**Fig. 3:**
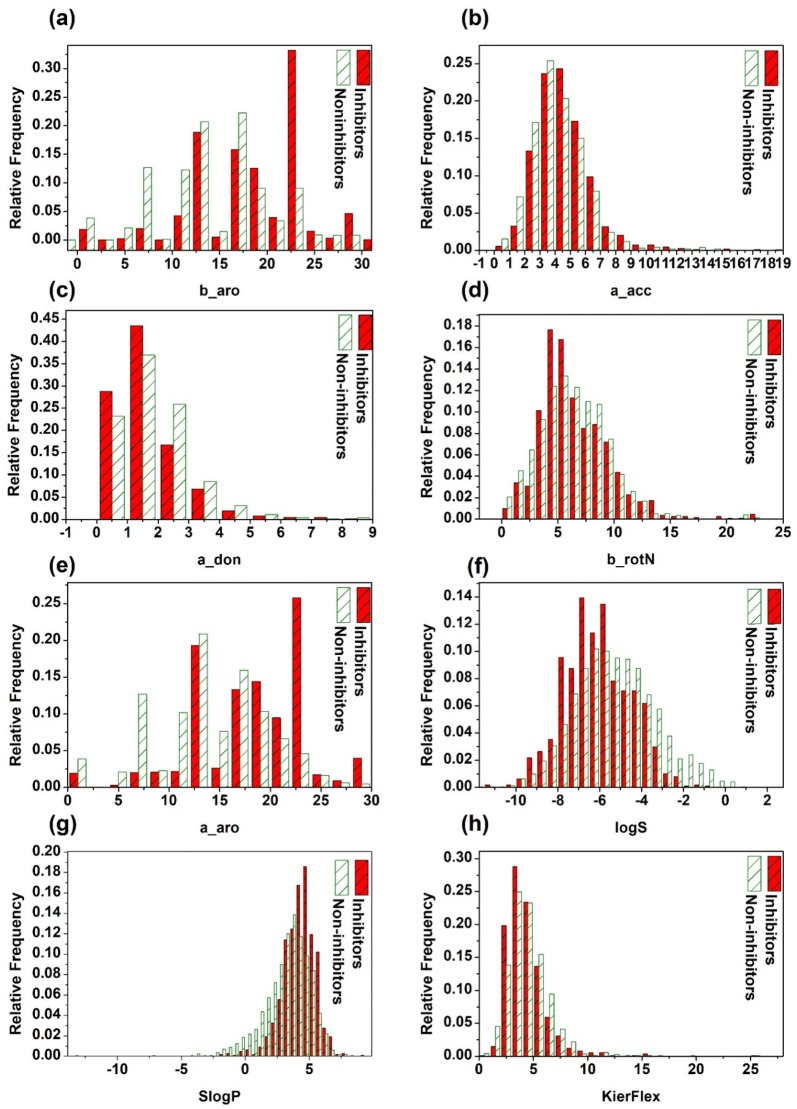
Distributions of the eight simple molecular descriptors, including SlogP, logS, a_acc, a_don, b_rotN, KierFlex, a_aro, and b_aro for the BCRP inhibitors and non-inhibitors

Various simple molecular descriptors have been used extensively in ADMET prediction and proven to be helpful [86], and they can characterize molecular properties from different aspects, such as hydrophobicity, flexibility, H-bonding ability, etc. It is interesting to analyze whether BCRP inhibitors and non-inhibitors can be distinguished by any of those molecular descriptors. Thus, we systematically explored the discrimination capability of eight molecular descriptors on BCRP inhibition. The examined descriptors include octanol–water partition coefficient (SlogP), aqueous solubility (logS), H-bond acceptor count (a_acc), H-bond donor count (a_don), flexible rotatable bond count (b_rotN), molecular flexibility index (KierFlex), aromatic atom count (a_aro), and aromatic bond count (b_aro). A non-parametric test method, known as Mann–Whitney U-test, was used to assess the significance of the difference between the means for inhibitors and non-inhibitors. Compared with Student’s t-test, Mann–Whitney U-test gains a competitive advantage because it does not require the assumption of normal distributions for the dataset. The distributions of these eight descriptors for the BCRP inhibitors and non-inhibitors are shown in Fig. 3. Besides, the means and associated *p*-values of the eight descriptors between the inhibitors and non-inhibitors are listed in Table 1.

**Table 1:** The mean values of eight simple descriptors for inhibitors and non-inhibitors and the associated *p*-values determined by Mann–Whitney U-test for each descriptor

Class	SlogP	logS	a_acc	a_don	b_rotN	KierFlex	a_aro	b_ar
Inhibitors	4.26	− 6.07	4.10	1.19	6.09	4.36	17.51	18.01
Non-inhibitors	3.29	− 5.25	3.70	1.41	5.88	4.53	13.46	13.73
*p*-value	2.16 × 10^−50^	4.63 × 10^−27^	1.32 × 10^−09^	1.12 × 10^−08^	6.38 × 10^−01^	3.14 × 10^−04^	6.90 × 10^−75^	5.90 × 10^−80^

In the eight studied descriptors, both SlogP and logS are related to the hydrophobicity and solubility of a molecule. As shown in Table 1, the mean values of SlogP for the inhibitors versus non-inhibitors are 4.26 and 3.29, respectively, and those of logS for the inhibitors versus non-inhibitors are − 6.07 and − 5.25, respectively. In other words, BCRP inhibitors are more hydrophobic and less soluble than non-inhibitors, which is consistent with the finding that the pharmacophore models for BCRP inhibitors contain hydrophobic features reported in the previous studies [11, 113]. By investigating the molecular descriptors that determine the interaction between BCRP and its inhibitors, Matsson et al. found that octanol–water partition coefficient at pH 7.4 (logD_7.4_) is an influential descriptor for the binding of inhibitors to BCRP [16]. The *p*-value for SlogP is 2.16 × 10^−50^ at the 95% confidence level, suggesting that the SlogP distributions for the inhibitors and non-inhibitors are remarkably different. Compared with the *p*-value for SlogP, that for logS is relatively higher (4.63 × 10^−27^ ).

As shown in Fig. 3 and Table 1, we can observe that the two H-bond features, a_acc and a_don, demonstrate relatively high impact on BCRP inhibition. The mean values of a_acc for the inhibitors and non-inhibitors are 4.10 and 3.70, respectively, and the associated *p*-value is 1.32 × 10^−9^. The mean values of a_don for the inhibitors and non-inhibitors are 1.19 and 1.41, respectively, and the associated *p*-value is 1.12 × 10^−8^. That is to say, BCRP inhibitors tend to contain more H-bond acceptors and less H-bond donors than BCRP non-inhibitors. This finding was supported by the previous studies that H-bond acceptors are quite important for BCRP inhibition [11, 16, 114].

The descriptors b_rotN and KierFlex are mainly used to describe the flexibility of a molecule. As can be seen from Table 1, the mean values of b_rotN for the two classes are very close and the associated *p*-value is 6.38 × 10^−1^ at the 95% confidence level, showing that the distribution of b_rotN for inhibitors and non-inhibitors is not statistically significant, and it is the same situation for KierFlex but its associated *p*-value is 3.14 × 10^−4^ at the 95% confidence level, demonstrating that the difference between the two distributions of KierFlex is statistically significant. However, as can be seen from Fig. 3 and Table 1, the distribution differences of these two properties between the two classes are not as significant as some other properties, such as hydrophobicity-related descriptors. The other two descriptors, a_aro and b_aro, are related to the aromaticity of a molecule. The mean values of a_aro for the inhibitors and non-inhibitors are 17.51 and 13.46, respectively, and the mean values of b_aro for the inhibitors and non-inhibitors are 18.01 and 13.73, respectively, which indicates that BCRP inhibitors exhibit stronger aromaticity than non-inhibitors. Our finding is in good agreement with the previous observations that aromaticity is an important molecular feature for BCRP inhibitors revealed by the computational model developed by Matsson and co-workers [115]. As can be seen from Fig. 3, the distributions of these two aromaticity-related descriptors differ notably between the two classes, and the associated *p*-values are 6.90 × 10^−75^ and 5.90 × 10^−80^, respectively, indicating that aromaticity is the best indicator to discriminate inhibitors from non-inhibitors. In summary, our findings imply that BCRP inhibitors are generally more hydrophobic and aromatic than non-inhibitors, which are consistent with the results reported by the previous studies [16, 73, 115, 116]. However, as shown in Fig. 3, for any descriptor, the distributions for the BCRP inhibitors and non-inhibitors still overlap largely, suggesting that any single molecular property cannot discriminate between BCRP inhibitors and non-inhibitors effectively.

### Feature selection based on rfSA

**Fig. 4:**
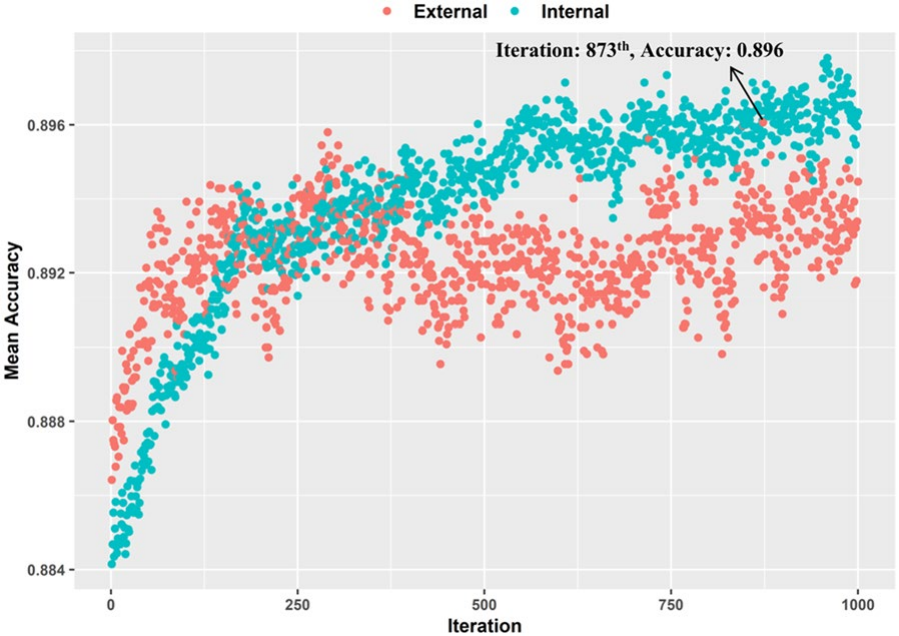
The feature selection process of molecular features through the rfSA method. Internal: four fifth of the training set in the fivefold cross-validation with five repetitions; External: one-fifth of the training set in the fivefold cross-validation with five repetitions

At first, a total of 1260 molecular features, including 379 molecular descriptors and 881 PubChem fingerprints, were calculated for each molecule in the final dataset. After eliminating the features with missing values and the redundant features, a smaller feature subset containing 382 molecular features was obtained and normalized. Then, a wrapper feature selection method known as rfSA was employed to identify the most representative features for QSAR modeling based on the training set. As shown in Fig. 4, the mean predictive accuracy of the external set in the fivefold cross-validation with five repetitions has reached the maximum at the 873th iteration (0.896). Therefore, the best iteration for the SA optimization was set to 873, and the SA algorithm at this iteration selected 144 features. As mentioned earlier, the selected 144 features were regarded as the optimal feature subset, where includes 65 molecular descriptors and 79 fingerprints. The detailed descriptions of these representative descriptors and fingerprints are listed in Additional file 1: Tables S3 and S4.

**Fig. 5:**
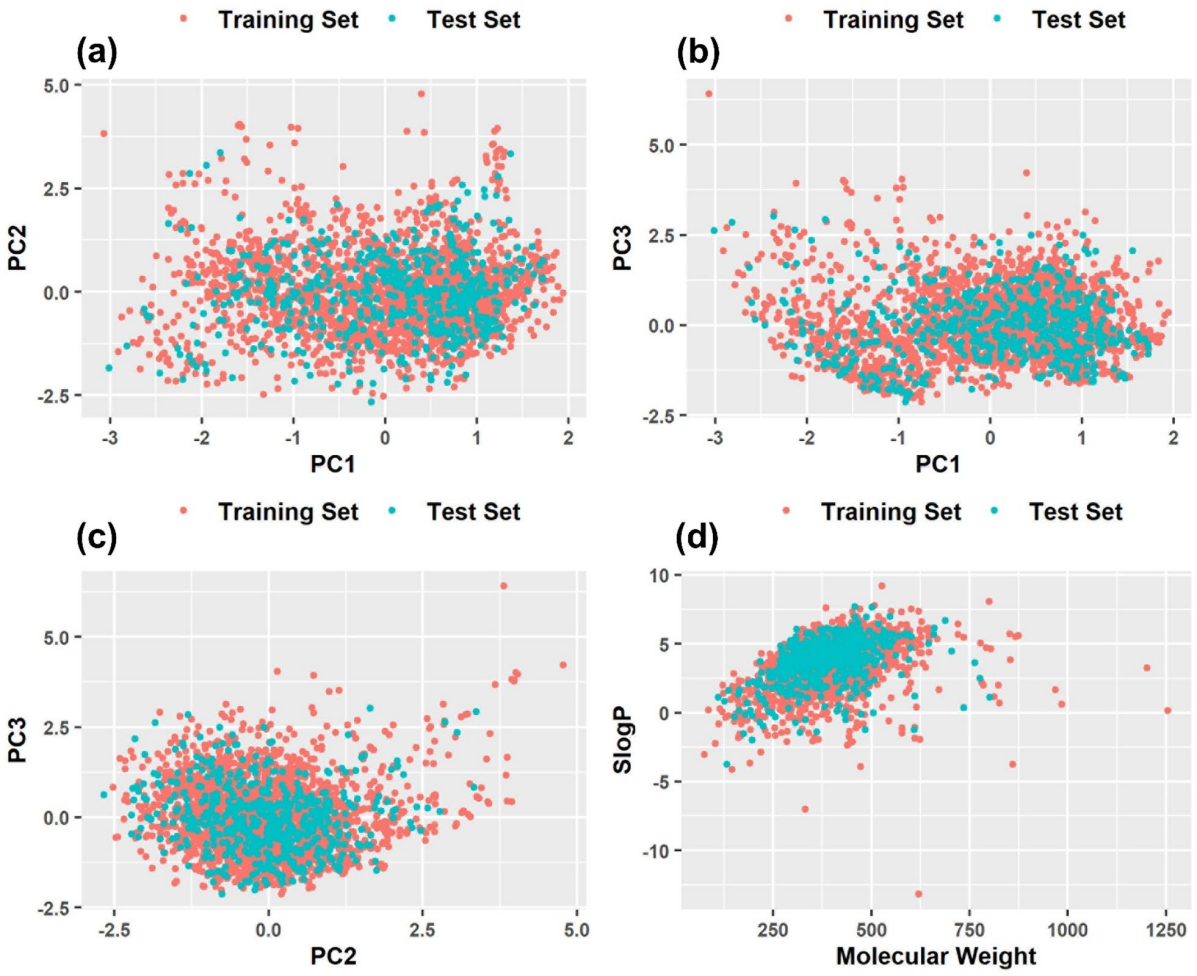
The chemical space distributions based on the principal components analysis (PCA): **a** The distributions of PC1 and PC2; **b** The distributions of PC1 and PC3; **c** The distributions of PC2 and PC3. **d** The chemical space defined by molecular weight as X-axis and SlogP as Y-axis

The chemical space distributions of the training and test sets were characterized by the scatter plots of the first three principal components derived from the principal component analysis (PCA) based on the 144 molecular features. Furthermore, the scattered distributions of molecular weight and SlogP [117] were also used to determine the chemical space distributions. As shown in Fig. 5a–d, the chemical space of the test set roughly falls within that of the training set, implying that it is feasible and reliable to use the test set to validate the prediction performance and generalization capability of the QSAR models developed from the training set.

### Comparison of various classification models for BCRP inhibition

Base on the 144 molecular features selected from the training set, seven ML methods, including one DL method (DNN), two ensemble learning methods (XGBoost and SGB) and four traditional ML methods (NB, k-NN, RLR, and SVM), were used to develop the QSAR models to distinguish BCRP inhibitors from non-inhibitors. In model development, the Bayesian optimization technique was employed to determine the optimal settings of hyper-parameter for all the methods. The statistical results for the random fivefold cross-validated training and test sets given by the optimized QSAR models are summarized in Table 2.

**Table 2:** Statistical results of the seven classification models based on 144 molecular features for the training (random fivefold cross-validation) and test sets

	Training set (random fivefold cross-validation)				Test set			
	GA	BA	MCC	AUC	GA	BA	MCC	AUC
SVM	0.902	0.893	0.793	0.947	0.911	0.905	0.812	0.958
DNN	0.894	0.892	0.780	0.950	0.907	0.904	0.806	0.960
XGBoost	0.902	0.894	0.793	0.956	0.891	0.883	0.770	0.957
SGB	0.901	0.894	0.792	0.952	0.886	0.879	0.759	0.958
RLR	0.875	0.872	0.740	0.932	0.873	0.867	0.734	0.936
k-NN	0.863	0.862	0.717	0.913	0.857	0.856	0.705	0.917
NB	0.826	0.834	0.654	0.898	0.780	0.793	0.572	0.888
Consensus1	0.902	0.893	0.793	NA	0.903	0.897	0.797	NA
Consensus2	0.901	0.895	0.793	0.956	0.909	0.903	0.808	0.963

*NA* not available

**Fig. 6:**
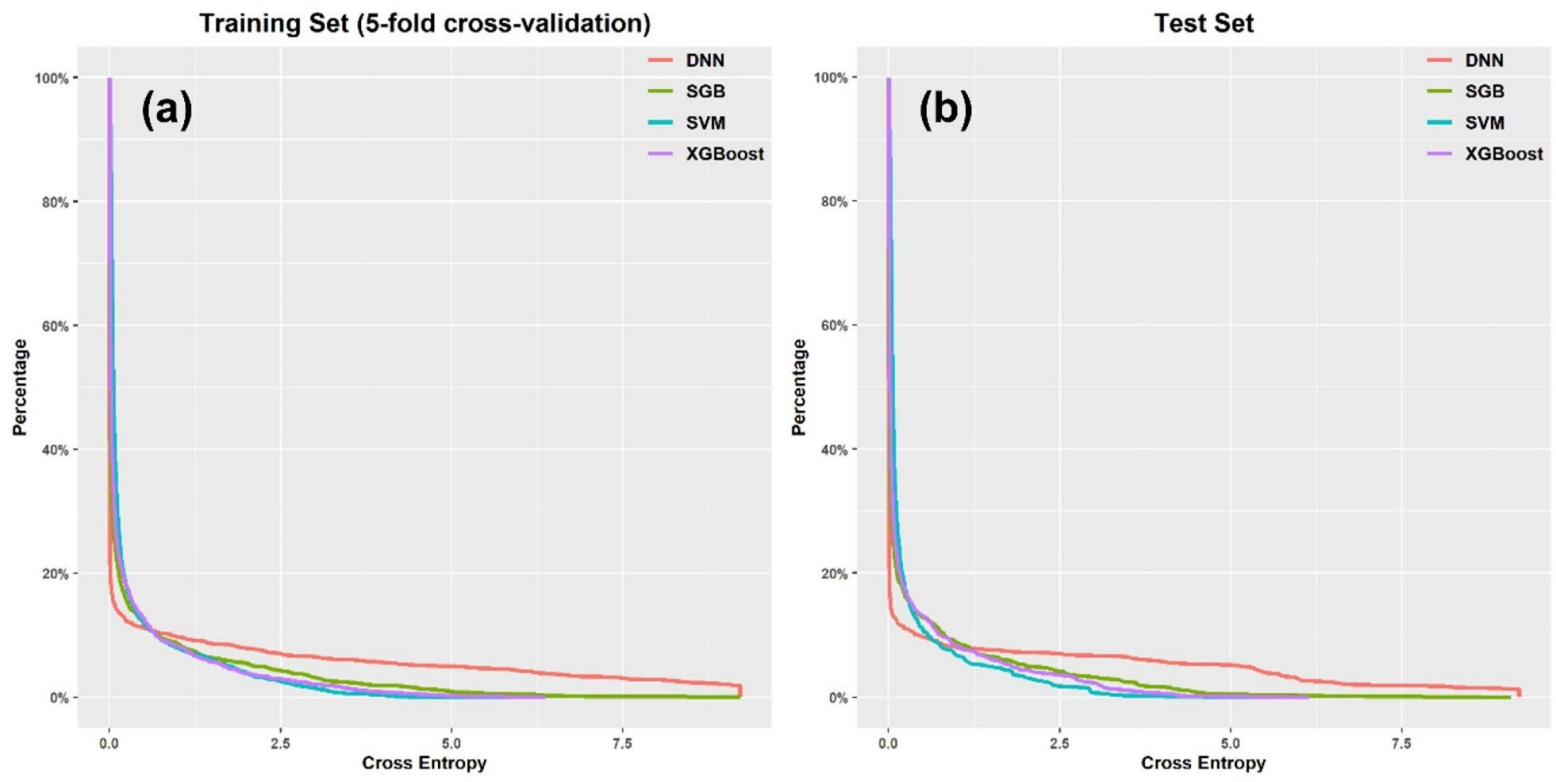
The residual (binary cross entropy) distribution plots of the **a** fivefold cross-validated training set and **b** test set (**b**) for the four well-performing QSAR models (DNN, SGB, XGBoost, and SVM). X axis represents the value of binary cross entropy, and Y axis represents the ratio of number of compounds with the residuals higher than a value to the total number of compounds in the training or test set

As shown in Table 2, it can be observed that four models (i.e., SVM, DNN, XGBoost, and SGB) give satisfactory statistical parameters for both the fivefold cross-validated training set and the test set. It is obvious that they have potential advantage over the other three traditional models (i.e., NB, RLR, k-NN) on the BCRP inhibition prediction in general. Here, SVM, a classical ML method, yields the best predictions for the test set when MCC and GA were used as the main criteria for the classification ability of models (MCC = 0.812 and GA = 0.911). Compared with GA, BA may give a more comprehensive consideration on sensitivity and specificity and it is capable of avoiding exaggerated assessment of model performance on an unbalanced data set. According to BA, the SVM model also gives the best prediction (BA = 0.905) on the test set. However, we found that the other three methods, including DNN, XGBoost, and SGB, showed similar performance to SVM (Table 2). Therefore, it is difficult to determine which model is the best only according to the confusion matrix based statistical parameters. In view of that, the residual distribution plot was used to further diagnose the quality of those four well-performing models (i.e., SVM, DNN, XGBoost, and SGB). As shown in Fig. 6, the residuals derived from the DNN model are larger than those derived from the other three models for both the fivefold cross-validated training set and test set, implying that the DNN model gives the worst class probability estimation for some compounds although it may still give correct classification for them. As shown in Fig. 6, the residual distribution of the XGBoost model is similar to that of the SVM model. However, it should be noted that, for those compounds with smaller residuals (smaller than 0.7), the DNN model gives the best class probability estimation and the other three models show similar performance. In conclusion, based on the statistical parameters and residual distributions, SVM, DNN and XGBoost were recommended to develop the classification models for BCRP inhibition, and among them, SVM is the best choice.

Notably, considerable efforts have been dedicated to explore the performance of DNN on QSAR modeling and many previous studies demonstrated that DNN is the best classifier compared with other conventional methods such as NB, RF, and SVM [32, 99, 118]. In this study, the DNN model is an outstanding but not the best classifier for BCRP inhibition prediction. As all we know, the DNN method can benefit from big data and is able to perform automatic representation learning from original feature space without the requirement of feature selection. In this study, it is possible that the performance of DNN was limited by the size of the dataset, the number of features used, and even the DNN implementation or hardware used. Finally, two consensus models (Consensus1 and Consensus2) were developed by combining the outputs from multiple models. Consensus1 is a class label consensus model based on the predictions given by the SVM, DNN, and XGBoost models, and Consensus2 is a class probability consensus model based on the predictions given by the SVM, DNN, and XGBoost models. All the contributions from the individual models were equal, and we could avoid overemphasizing any ML approach. As shown in Table 2, the two consensus models did not improve the predictivity obviously. However, the class probability consensus model gives the best AUC values for both the fivefold cross-validated training set (AUC = 0.956) and test set (AUC = 0.963). It could be found that the class probability consensus model is slightly better than the class label consensus model.

As described earlier, in addition to the evaluation for the random fivefold cross-validated training set, we also performed a cluster fivefold cross-validation for the training set to check the predictive capacity of the well-established model for the unseen compounds. A threshold value of 0.7 for complete linkage clustering identified 213 different compound clusters and the random distribution of them across fivefolds results in the numbers of compounds for different folds are 445, 447, 452, 442, and 454, respectively. Based on the cluster fivefold cross-validated training set, the seven ML methods were employed to developed the classification models with the same hyper-parameters determined in the random fivefold cross-validation and the optimal feature subsets determined by rfSA. As shown in Table 3, the four ML approaches (i.e., SVM, DNN, XGBoost, and SGB) that have better performance for the random fivefold cross-validated training set also give satisfactory predictions to the clustered fivefold cross-validated training set. Moreover, the SVM model gives the best MCC value of 0.810 to the cluster fivefold cross-validated training set, illustrating that the models developed in this study can also be used to predict the compounds with new scaffolds. Briefly, the SVM model gives the best predictions to both the training and test set. The AD statistical results defined by the non-parametric probability density distribution-based method shown in Table 4 illustrate that only 11 compounds are outside the AD. The AD coverages for the training and test sets are 100% and 98%, respectively, indicating that the predictions of the test set are reliable.

**Table 3:** Statistical results of the seven classification models for the cluster fivefold cross-validated training set

	Training set (cluster fivefold cross-validation)			
	GA	BA	MCC	AUC
SVM	0.910	0.901	0.810	0.954
DNN	0.905	0.897	0.794	0.949
XGBoost	0.905	0.897	0.800	0.959
SGB	0.901	0.892	0.791	0.956
RLR	0.879	0.876	0.748	0.935
k-NN	0.867	0.863	0.722	0.918
NB	0.819	0.823	0.634	0.896

**Table 4:** The number of compounds that are inside and outside the AD determined by the non-parametric probability density distribution-based method in the training and test sets

Dataset	Inside AD		Outside AD		AD coverage (%)
	N1^a^	N2^b^	N1^a^	N2^b^	
Training set	879	1361	0	0	100
Test set	219	340	1	10	98

^a^N1: the number of inhibitors

^b^N2: the number of non-inhibitors

### Comparison with published models and interpretation of models

In order to further estimate the prediction capacities of the built models, the performance of our models was compared with those of some models reported in the previous studies. As shown in Table 5, a majority of the reported classification models for differentiating BCRP inhibitors and non-inhibitors were developed using conventional ML methods such as NB, k-NN, SVM and RF based on relatively small datasets less than 1000 compounds. The GA values of the published models for the test or external set varied from 0.66 to 0.878 and the MCC values varied from 0.29 to 0.73. In the reported classification models, it seems that the classifier reported by Montanari et al. gives the best performance, indicated by a GA value of 0.919 for the tenfold cross-validated training set. Unfortunately, this classifier gives an unsatisfactory AUC value of 0.854 for the tenfold cross-validated training set and it was not validated by any test set. Compared with the published classification models, the models developed in this study show better prediction capability. The GA and MCC values given by the SVM model for the test set are 0.911 and 0.812, respectively, and the AUC value given by the Consensus2 model is 0.963.

**Table 5:** The reported classification models for BCRP inhibitors and non-inhibitors

Year	Data size	Data set		Method	Descriptors	Model validation	Statistical results	Refs.
		Training	Test					
2007	123	80	43	OPLS-DA	Descriptors from SELMA software package	Y-rand	GA_TE_ = 0.79	Matsson et al. [16]
2009	122	83	39	PLS-DA	Descriptors from DragonX version 3.0	Y-rand	^a^NA	Matsson et al. [115]
2013	109	30	79	Pharmacophore modeling	NA	NA	MCC_TE_ = 0.29, GA_TE_ = 0.66	Pan et al. [11]
2013	203	124	79	NB	ECFP_6, FCFP_6 fingerprints	LOO CV	AUC_TR(LOO CV)_ = 0.795, MCC_TE_ = 0.69	Pan et al. [11]
2013	382	382	NA	SVM, k-NN, RF, and consensus modeling	Dragon, MOE descriptors	Fivefold CV, Y-rand	BA_TR(fivefold cv)_ = 0.83 ± 0.04 (Consensus)	Sedykh et al. [121]
2014	275	96	Test: 32, external set: 147	ensembles of ANN, ensembles of SVM	Descriptors from ADMET Modeler	NA	GA_TE_ = 0.87, GA_External_ = 0.67 (ensembles of ANN)	Eric et al. [122]
2014	780	780	NA	NB	ECFP_6 fingerprints	Tenfold CV	GA_TR(tenfold CV)_ = 0.919, AUC_TR(tenfold cv)_ = 0.854	Montanari et al. [20]
2015	394	197	Test: 99, external set: 98	SVM, k-NN, ANN, and Consensus Modeling	Dragon descriptors	NA	GA_TE_ = 0.878, MCC_TE_ = 0.73; GA_External_ = 0.745, MCC_External_ = 0.46 (ANN)	Belekar et al. [21]
2016	^a^NA	NA	NA	GTM-kNNd, GTM-Bayes, RF, SVM, and k-NN	MOE descriptors	Fivefold CV with five repetitions	NA	Gimadiev et al. [123]
2017	978	978	NA	NB, LR, SVM, and RF	MACCS, Morgan, ECFP8 fingerprints, VolSurf descriptors	Tenfold CV, leave-sources-out validation	MCC_TR(tenfold CV)_ = 0.65, AUC_TR(tenfold CV)_ = 0.90 (LR)	Montanari et al. [22]
2019	2799	2240	559	NB, LR, SVM, k-NN, XGBoost, SGB, DNN and consensus modeling	MOE descriptors and Pubchem fingerprints	Fivefold CV	MCC_TE_ = 0.812, AUC_TE_ = 0.958, GA_TE_ = 0.911, BA_TE_ = 0.905 (SVM)	This study

Mean ± st.dev across fivefold CV

*TR* training set, *TE* test set, *OPLS-DA* orthogonal partial least-squares projection to latent structures discriminant analysis, *NA* not available, *GA* global accuracy, *Y-Rand* Y-Randomization test, *PLS-DA* partial least-squares projection to latent structures discriminant analysis, *NB* Naive Bayes, *LOO CV* leave-one-out cross-validation, *AUC* the area under the receiver operating characteristic curve, *MCC* Matthews correlation coefficient, *SVM* support vector machine, *k-NN* k-nearest neighbors, *RF* random forest, *CV* cross-validation, *BA* balanced accuracy, *ANN* artificial neural networks, *GTM* generative topographic mapping, *LR* logistic regression

There are many models developed based on different methods or descriptors, and we only extracted the best statistical results for the test set or cross-validation

^a^The exact values are not available in the publication

**Fig. 7:**
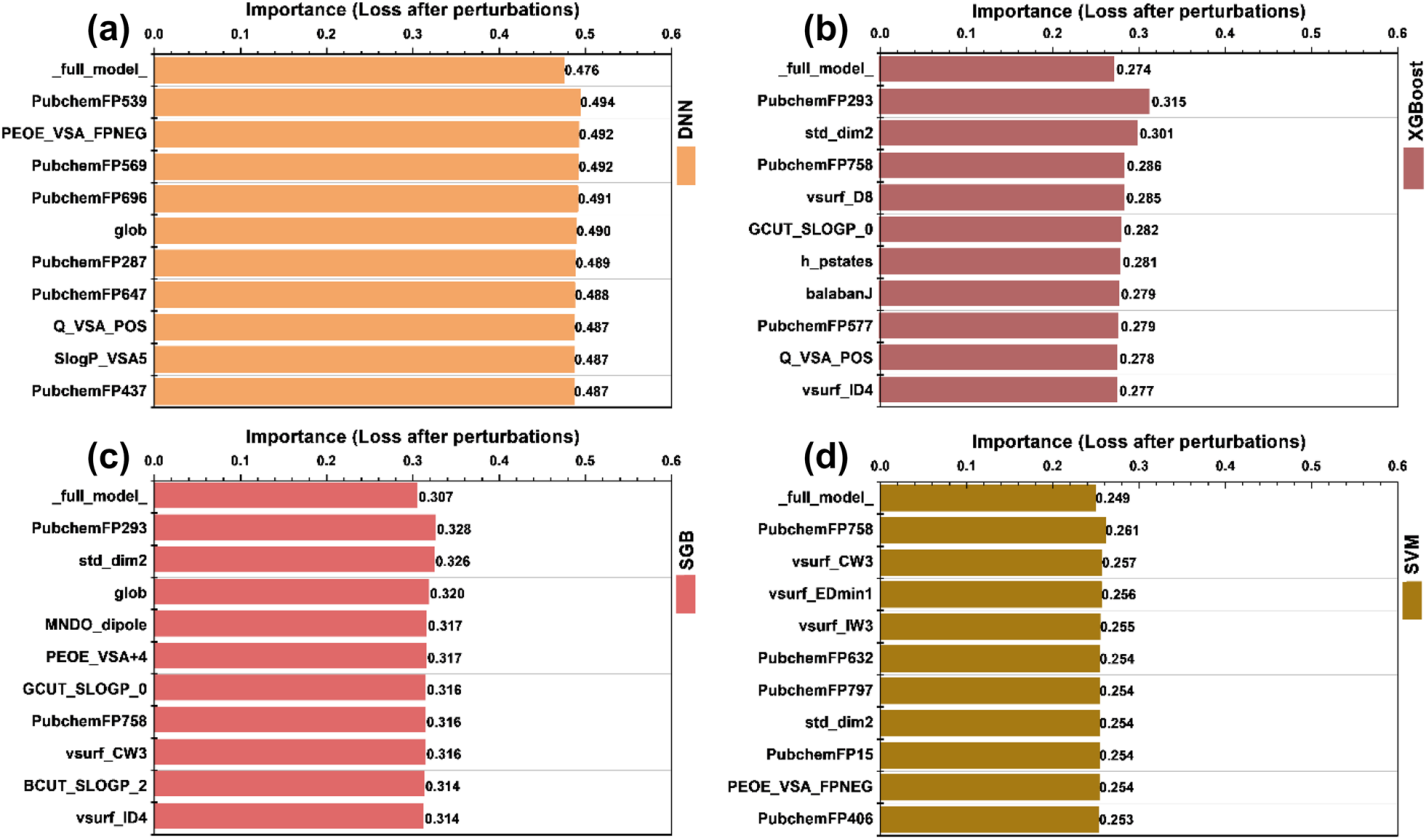
The top ten descriptors/fingerprints for the **a** DNN, **b** XGBoost, **c** SGB and **d** SVM models identified by the perturbation-based model-agnostic method with 10 repetitions. The term “full_model_” denotes the estimation of a model performance on the test set based on the cross entropy loss function

Model interpretation is a recommended procedure during QSAR modeling in the OECD principles [100]. Here a perturbation-based model-agnostic method was employed to identify and analyze the important features for the SVM, DNN, XGBoost and SGB models. The top ten descriptors for each model are shown in Fig. 7. It can be observed that the DNN model has the highest average binary cross entropy for the test set (0.476), and the SVM model has the lowest value (0.249). Those results are in good agreement with the observed residual distributions shown in Fig. 6 that the residual distribution curve of the DNN model is the highest, to some extent, among the four curves and that of the SVM model is the lowest. Another interesting finding is that different important features were identified for different models, indicating that different methods handle the features in a very varying ways and it potentially depends on the basic principle of approach. Besides, it is quite possible that a set of important descriptors/fingerprints for a well-performing model may be not suitable for another model.

However, we found that some descriptors/fingerprints related to molecular lipophilicity (BCUT_SLOGP_2, GCUT_SLOGP_0, and SlogP_VSA5), molecular surface area, volume and shape (std_dim2, glob, and vsurf_descriptors) and electronic properties (PEOE_VSA_ descriptors and Q_VSA_POS) were captured by most well-performing models. The importance of molecular lipophilicity descriptors in the development of QSAR models to distinguish BCRP inhibitors from non-inhibitors has been highlighted by the previous studies [8, 15, 21]. Moreover, according to the two-step mechanism for the inhibition of BCRP proposed by Matsson et al., high lipophilicity is a prerequisite for an inhibitor to reach the binding site of BCRP in the first step, and then geometrical complementary, H-bonding and π–π interactions, which can be described by the shape, H-bond and electronic descriptors (such as std_dim2, PEOE_VSA_FPNEG and PEOE_VSA+4) [115], determine the binding of inhibitors to BCRP in the second step.

It has been reported that the addition of fingerprints can significantly improve the prediction accuracy for many QSAR models [86, 88]. As shown in Fig. 7, some important features are fingerprints. The fingerprint PubchemFP758 (Cc1c(N)cccc1) was identified by three well-performing models (XGBoost, SGB and SVM) and PubchemFP293 (C-S) was identified by two well-performing models (XGBoost and SGB). We then analyzed the frequencies of these two important fingerprints, and found that their distributions for the inhibitors and non-inhibitors are quite different: 48.4% inhibitors (531 out of 1098) and 20.1% non-inhibitors (342 out of 1701) contain PubChem758, respectively, and 47.2% non-inhibitors (803 out of 1701) and 6.2% inhibitors (68 out of 1098) contain PubChem293, respectively. The frequency analysis of PubchemFP758 that is a *o*-Toluidine fragment implies that BCRP inhibitors are more aromatic than non-inhibitors, which is in line with the results reported by Sjöstedt et al. and Matsson et al. that aromaticity is an important feature of BCRP inhibitors [73, 115]. PubChem293 contains a sulfur atom, which is supported by the results reported by Montanari and Ecker that the presence of a sulfur atom is unfavorable to BCRP inhibition due to its steric hindrance in the binding site [20].

### The important fragments identified by the IG method

As described in “Materials and methods”, three kinds of fragments were generated for all the compounds and the frequency values of generated fragments in the inhibitor class and non-inhibitor class were calculated. If the occurrence of a fragment is more frequent in the inhibitor class than in non-inhibitor class, this fragment was considered as an important fragment for the inhibitor class, and it has positive contribution to BCRP inhibition and vice versa. The IG value was also calculated for all the generated fragments to identify the important structural fragments to BCRP inhibition.

**Fig. 8:**
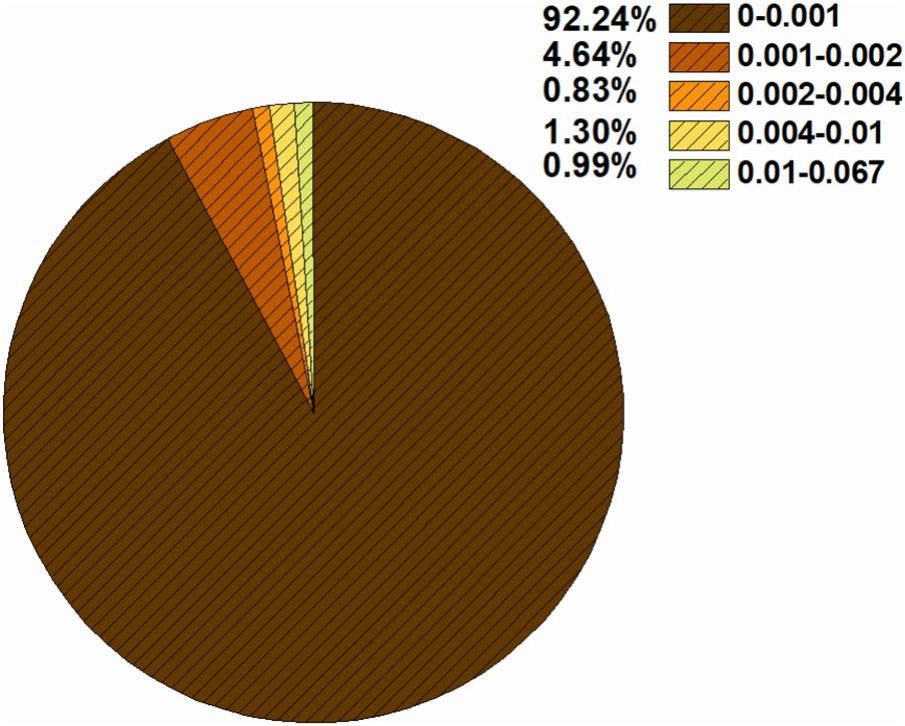
The information gain (IG) value distributions of the generated fragments

As shown in Fig. 8, the IG values of the fragments range from 0 to 0.067, and most fragments have relatively low IG values. Analyses of the IG values and frequencies of the fragments show that 12 positive fragments are responsible for BCRP inhibition (Table 6). All of them have high IG values and their frequencies for the inhibitor and non-inhibitor classes are obviously different. Similarly, 11 negative fragments were identified to be responsible for BCRP non-inhibition (Table 7).

**Table 6:** The identified fragments by the information gain (IG) method couple with frequency analysis for BCRP inhibitors

Fragment	Name	Count1^a^	Count2^b^	F1^c^	F2^d^	IG
	Quinazoline	175	14	2.3604	0.1219	0.0665
	*N*,2-diphenylquinazolin-4-amine	92	0	2.5492	0.0000	0.0456
	Pyrazolo[1,5-a]pyrimidine	85	8	2.3299	0.1415	0.0299
	4H-chromene	104	23	2.0875	0.2980	0.0263
	2-phenyl-4H-chromene	55	3	2.4173	0.0851	0.0216
	2H-tetrazole	65	12	2.1519	0.2564	0.0177
	Piperazine	90	30	1.9119	0.4114	0.0171
	*N*,2-diphenylpyrido[2,3-d]pyrimidin-4-amine	31	0	2.5492	0.0000	0.0151
	2,3,4,9-tetrahydro-1H-pyrido[3,4-b]indole	29	0	2.5492	0.0000	0.0141
	(R)-2-benzyl-1-phenyl-2,3,4,9-tetrahydro-1H-pyrido[3,4-b]indole	27	0	2.5492	0.0000	0.0131
	*N*-benzyl-2-(2-phenyl-2H-tetrazol-5-yl)aniline	41	5	2.2721	0.1789	0.0131
	Pyrido[2,3-d]pyrimidine	32	2	2.3992	0.0968	0.0122

^a^Count1 denotes the number of inhibitors containing the fragment

^b^Count2 denotes the number of non-inhibitors containing the fragment

^c^F1 denotes the fragment frequency in the inhibitor class

^d^F2 denotes the fragment frequency in the non-inhibitor class

**Table 7:** The identified fragments by the information gain (IG) method couple with frequency analysis for BCRP non-inhibitors

Fragment	Name	Count1^a^	Count2^b^	F1^c^	F2^d^	IG
	1H-pyrazole	1	67	0.0375	1.6213	0.0153
	Thiazolidine	0	56	0.0000	1.6455	0.0146
	Thiophene	16	118	0.3044	1.4490	0.0133
	Morpholine	0	51	0.0000	1.6455	0.0132
	4H-1,2,4-triazole	0	48	0.0000	1.6455	0.0125
	Thiazole	3	62	0.1177	1.5696	0.0113
	Benzo[d]thiazole	1	50	0.0500	1.6132	0.0109
	1,3,4-thiadiazole	0	36	0.0000	1.6455	0.0093
	Hexahydropyrimidine	2	44	0.1108	1.5740	0.0081
	Piperidine	3	46	0.1561	1.5448	0.0075
	Pyrrolidine	1	36	0.0689	1.6010	0.0074

^a^Count1 denotes the number of inhibitors containing the fragment

^b^Count2 denotes the number of non-inhibitors containing the fragment

^c^F1 denotes the fragment frequency in the inhibitor class

^d^F2 denotes the fragment frequency in the non-inhibitor class

As shown in Tables 6 and 7, it can be observed that those positive fragments contain more abundant nitrogen atoms, and 6 of them contain at least three nitrogen atoms (No. 2, 3, 6, 8, 11, and 12). However, most negative fragments have only one or two nitrogen atoms or even do not contain any nitrogen atom. In addition, it is obvious that the positive fragments are more planar. According to the previous studies, it is suggested that the abundance of nitrogen atoms and molecular planarity have a high impact on BCRP inhibition [16, 20]. Among the 12 positive fragments, No. 2 fragment (*N*,2-diphenylquinazolin-4-amine) has been reported to be favorable to BCRP inhibition [119]. In addition, It is obvious that almost all the negative fragments, with the exception of No. 19, contain only one individual ring in their structures, but most positive fragments contain at least two individual rings. This is in good agreement with the results that BCRP inhibitors tend to have more rings than non-inhibitors [73]. The 11 fragments shown in Table 7 have negative contributions to BCRP inhibition. It is obvious that many fragments do not contain planar components, and they have unfavorable effects on BCRP inhibition because molecular planarity is critical to BCRP inhibition as mentioned above. Many negative fragments contain a sulfate group, including No. 14, 15, 18, 19, and 20. According to the previous study, it is noted that the presence of sulfate is unfavorable to BCRP inhibition because sulfate is bulky, which may lead to some kinds of steric hindrance in the binding site [20].

Generally speaking, the positive fragments are larger than negative fragments, and contain more rings and nitrogen atoms. The negative fragments are less planar and contain more sulfate groups.

### Analysis of the misclassified compounds

As discussed above, the SVM model developed based on the selected features from the training set yields the best predictions for the test set, but it still misclassified 50 compounds in the test set. These misclassified compounds include 27 false negatives and 23 false positives. The detailed descriptions of the misclassified molecules are listed in Additional file 1: Table S5.

First, we analyzed the scaffolds of the 50 misclassified compounds. Each compound was decomposed into three kinds of structural scaffolds including Ring Assemblies, Bridge Assemblies, and BemisMurcko Assemblies. For the generated fragments whose counts in the misclassified compounds are equal or larger than 2 were kept and their occurrence ratios were calculated. In the same way, their counts and occurrence ratios were also calculated for the training and test sets (Table 8). Most of those representative fragments contain hetero-cycles with nitrogen, oxygen or sulfur atom. These fragments are not abundant in the whole dataset (average occurrence ratio in the dataset is 3.65%) and some fragments in the test set are not enriched in the training set (fragments No. 26 and 36). It is quite possible that the SVM model could not give accurate predictions to those infrequent fragments.

**Table 8:** The representative fragments whose counts are equal or larger than 2 in the 50 misclassified compounds

Fragment	Name	Count1^a^	Count2^a^	Count3^a^	OR1^a^ (%)	OR2^a^ (%)	OR3^a^ (%)
	Pyridine	125	43	5	5.58	7.69	10.00
	Tetrahydro-2H-pyran	37	6	3	1.65	1.07	6.00
	Adamantane	6	3	2	0.27	0.54	4.00
	(E)-prop-1-ene-1,3-diyldibenzene	58	16	3	2.59	2.86	6.00
	1H-indole	82	25	3	3.66	4.47	6.00
	4H-chromene	110	17	4	4.91	3.04	8.00
	Furan	175	37	2	7.81	6.62	4.00
	Piperazine	98	22	3	4.38	3.94	6.00
	Quinazoline	150	39	2	6.70	6.98	4.00
	Quinoline	58	16	3	2.59	2.86	6.00
	Thiophene	109	25	3	4.87	4.47	6.00
	1,2,3,4-tetrahydroi	56	14	3	2.50	2.50	6.00
	2-(4-((quinolin-3-ylmethyl)amino)phenethyl)-1,2,3,4-tetrahydroi	0	2	2	0.00	0.36	4.00

^a^Count1, 2, and 3 represent the number of the training compounds, testing compounds and misclassified compounds containing the fragment, respectively, and OR1, 2 and 3 represent the occurrence ratio of the fragment in the training compounds, testing compounds and misclassified compounds, respectively

Among the 50 misclassified compounds, except compound No. 221 that only contains a single ring, most of them have complicated structures. It is quite possible that some specific groups in those complex compounds need specific spatial arrangements to form favorable interaction with BCRP, which may not be well characterized by traditional descriptors or fingerprints [29]. Furthermore, in this study, the reported experimental data were binarized according to a predefined threshold, and therefore the compounds with experimental data close to the threshold may be more likely to be misclassified than those with experimental data far from the threshold, which has been well explained by Wu et al. [120]. In addition, existence of a few activity cliffs (similar structures but different activities) that distort the models may lead to misclassifications [29]. For example, in the 48 misclassified compounds, a pair of compounds (compounds No. 90 and 1360) may be a pair of activity cliffs (Tanimoto similarities based on the 881 PubChem fingerprints are 0.923).

Here, five of the 27 misclassified inhibitors (compounds No. 3, No. 472, No. 1606, No. 1657, and No. 1806) contain a sulfate group, which is unfavorable to BCRP inhibition as mentioned above [20]. Thus, this is one of the potential reasons why they were easily predicted as non-inhibitors. Two misclassified non-inhibitors (compounds No. 237 and No. 239) have abundant methoxyl (–OCH_3_) groups. The methoxyl groups observed in most QSAR studies could be interpreted as favorable H-bond acceptors [8], which may have positive contributions to BCRP inhibition [11, 16, 114].

## Conclusion

As an important efflux transporter, BCRP plays a crucial role in drug metabolism, multi-drug resistance to chemotherapeutic agents, and drug–drug interactions. It is an increasingly important area to develop computational models with high reliability and robustness to predict BCRP inhibition. In this study, we reported an extensive and structurally diverse BCRP inhibition dataset, where includes 1098 BCRP inhibitors and 1701 non-inhibitors. The relationships between the eight simple molecular descriptors and BCRP inhibition were examined. It is observed that BCRP inhibitors were characterized by higher hydrophobicity and aromaticity. The optimal feature subset of 144 molecular features was determined based on a wrapper feature selection named rfSA. Then, based on the extensive BCRP inhibition dataset and the optimal subset, seven ML approaches, including one DL method: DNN, two ensemble learning methods: SGB and XGBoost, and four classical methods: NB, weighted k-NN, RLR, and SVM, were used to develop a number of accurate classification models. Bayesian optimization technique was used to select the optimal hyper-parameters for all the models. This is the first attempt that the new AI methods (i.e., XGBoost and DNN) were used to predict BCRP inhibition. The statistical results indicated that one conventional ML method (SVM) and two new ML methods (XGBoost and DNN) outperformed the other three traditional ML methods, and among them SVM yielded the best prediction capability. Then, a perturbation-based method was used to interpret our models and analyze the representative features for different models. In addition, the favorable and unfavorable fragments to BCRP inhibition identified by information gain (IG) method along with frequency analysis were then analyzed. The results may provide some valuable information for the design and discovery of BCRP inhibitors.

## Supplementary information

**Additional file 1: Table S1**. The detailed descriptions of the final dataset containing 1098 BCRP inhibitors and 1701 BCRP non-inhibitors. **Table S2**. The searching space of the Bayesian optimization and the optimal hyper-parameters determined by the Bayesian optimization for all the studied models; **Table S3.** The detailed descriptions of the 65 representative descriptors chosen by rfSA; **Table S4.** The detailed descriptions of the 79 representative fingerprints chosen by rfSA; **Table S5**. The detailed descriptions of the misclassified molecules given by the SVM mode1.

**Additional file 2.** The R source code that implements the workflow.

## References

[1] Ni Z, Bikadi Z, Rosenberg MF, Mao Q (2010). Structure and function of the human breast cancer resistance protein (BCRP/ABCG2). Curr Drug Metab.

[2] Mao QC, Unadkat JD (2005). Role of the breast cancer resistance protein (ABCG2) in drug transport. AAPS J.

[3] Doyle LA, Ross DD (2003). Multidrug resistance mediated by the breast cancer resistance protein BCRP (ABCG2). Oncogene.

[4] Mao Q, Unadkat JD (2015). Role of the breast cancer resistance protein (BCRP/ABCG2) in drug transport-an update. AAPS J.

[5] Garg P, Dhakne R, Belekar V (2015). Role of breast cancer resistance protein (BCRP) as active efflux transporter on blood–brain barrier (BBB) permeability. Mol Divers.

[6] Szakacs G, Varadi A, Oezvegy-Laczka C, Sarkadi B (2008). The role of ABC transporters in drug absorption, distribution, metabolism, excretion and toxicity (ADME-Tox). Drug Discov Today.

[7] Krishnamurthy P, Schuetz J (2006). Role of ABCG2/BCRP in biology and medicine. Annu Rev Pharmacol Toxicol.

[8] Nicolle E, Boccard J, Guilet D, Dijoux-Franca M-G, Zelefac F, Macalou S, Grosselin J, Schmidt J, Carrupt P-A, Di Pietro A, Boumendjel A (2009). Breast cancer resistance protein (BCRP/ABCG2): new inhibitors and QSAR studies by a 3D linear solvation energy approach. Eur J Pharm Sci.

[9] Kruijtzer CMF, Beijnen JH, Rosing H, Huinink WWT, Schot M, Jewell RC, Paul EM, Schellens JHM (2002). Increased oral bioavailability of topotecan in combination with the breast cancer resistance protein and P-glycoprotein inhibitor GF120918. J Clin Oncol.

[10] Stewart CF, Leggas M, Schuetz JD, Panetta JC, Cheshire PJ, Peterson J, Daw N, Jenkins JJ, Gilbertson R, Germain GS, Harwood FC, Houghton PJ (2004). Gefitinib enhances the antitumor activity and oral bioavailability of irinotecan in mice. Cancer Res.

[11] Pan Y, Chothe PP, Swaan PW (2013). Identification of novel breast cancer resistance protein (BCRP) inhibitors by virtual screening. Mol Pharm.

[12] Pick A, Mueller H, Mayer R, Haenisch B, Pajeva IK, Weigt M, Boenisch H, Mueller CE, Wiese M (2011). Structure-activity relationships of flavonoids as inhibitors of breast cancer resistance protein (BCRP). Bioorg Med Chem.

[13] Saito H, Hirano H, Nakagawa H, Fukami T, Oosumi K, Murakami K, Kimura H, Kouchi T, Konomi M, Tao E, Tsujikawa N, Tarui S, Nagakura M, Osumi M, Ishikawa T (2006). A new strategy of high-speed screening and quantitative structure-activity relationship analysis to evaluate human ATP-binding cassette transporter ABCG2-drug interactions. J Pharmacol Exp Ther.

[14] Pick A, Mueller H, Wiese M (2008). Structure–activity relationships of new inhibitors of breast cancer resistance protein (ABCG2). Bioorg Med Chem.

[15] Zhang SZ, Yang XN, Coburn RA, Morris ME (2005). Structure activity relationships and quantitative structure activity relationships for the flavonoid-mediated inhibition of breast cancer resistance protein. Biochem Pharmacol.

[16] Matsson P, Englund G, Ahlin G, Bergstrom CAS, Norinder U, Artursson P (2007). A global drug inhibition pattern for the human ATP-binding cassette transporter breast cancer resistance protein (ABCG2). J Pharmacol Exp Ther.

[17] Gandhi YA, Morris ME (2009). Structure–activity relationships and quantitative structure–activity relationships for breast cancer resistance protein (ABCG2). AAPS J.

[18] Ishikawa T, Hirano H, Saito H, Sano K, Ikegami Y, Yamaotsu N, Hirono S (2012). Quantitative structure–activity relationship (QSAR) Analysis to predict drug–drug interactions of ABC transporter ABCG2. Mini-Rev Med Chem.

[19] Nicolle E, Boumendjel A, Macalou S, Genoux E, Ahmed-Belkacem A, Carrupt PA, Di Pietro A (2009). QSAR analysis and molecular modeling of ABCG2-specific inhibitors. Adv Drug Deliv Rev.

[20] Montanari F, Ecker GF (2014). BCRP inhibition: from data collection to ligand-based modeling. Mol Inform.

[21] Belekar V, Lingineni K, Garg P (2015). Classification of breast cancer resistant protein (BCRP) inhibitors and non-inhibitors using machine learning approaches. Comb Chem High Throughput Screen.

[22] Montanari F, Cseke A, Wlcek K, Ecker GF (2017). Virtual screening of DrugBank Reveals two drugs as new BCRP inhibitors. Slas Discov.

[23] Ryu JY, Kim HU, Lee SY (2018). Deep learning improves prediction of drug–drug and drug–food interactions. Proc Natl Acad Sci.

[24] Munir K, Elahi H, Ayub A, Frezza F, Rizzi A (2019). Cancer diagnosis using deep learning: a bibliographic review. Cancers.

[25] Ma J, Sheridan RP, Liaw A, Dahl GE, Svetnik V (2015). Deep neural nets as a method for quantitative structure–activity relationships. J Chem Inf Model.

[26] Klambauer G, Unterthiner T, Mayr A, Hochreiter S (2017). DeepTox: toxicity prediction using deep learning. Toxicol Lett.

[27] Wu Z, Ramsundar B, Feinberg EN, Gomes J, Geniesse C, Pappu AS, Leswing K, Pande V (2018). MoleculeNet: a benchmark for molecular machine learning. Chem Sci.

[28] Altae-Tran H, Ramsundar B, Pappu AS, Pande V (2017). Low data drug discovery with one-shot learning. ACS Cent Sci.

[29] Lei T, Chen F, Liu H, Sun H, Kang Y, Li D, Li Y, Hou T (2017). ADMET evaluation in drug discovery. Part 17: development of quantitative and qualitative prediction models for chemical-induced respiratory toxicity. Mol Pharm.

[30] Lei T, Li Y, Song Y, Li D, Sun H, Hou T (2016). ADMET evaluation in drug discovery: 15. Accurate prediction of rat oral acute toxicity using relevance vector machine and consensus modeling. J Cheminform.

[31] Sheridan RP, Wang WM, Liaw A, Ma J, Gifford EM (2016). Extreme gradient boosting as a method for quantitative structure–activity relationships. J Chem Inf Model.

[32] Korotcov A, Tkachenko V, Russo DP, Ekins S (2017). Comparison of deep learning with multiple machine learning methods and metrics using diverse drug discovery data sets. Mol Pharm.

[33] Aliper A, Plis S, Artemov A, Ulloa A, Mamoshina P, Zhavoronkov A (2016). Deep learning applications for predicting pharmacological properties of drugs and drug repurposing using transcriptomic data. Mol Pharm.

[34] Gawehn E, Hiss JA, Brown JB, Schneider G (2018). Advancing drug discovery via GPU-based deep learning. Expert Opin Drug Discov.

[35] D’Cunha R, Bae S, Murry DJ, An G (2016). TKI combination therapy: strategy to enhance dasatinib uptake by inhibiting Pgp- and BCRP-mediated efflux. Biopharm Drug Dispos.

[36] Elsby R, Fox L, Stresser D, Layton M, Butters C, Sharma P, Smith V, Surry D (2011). In vitro risk assessment of AZD9056 perpetrating a transporter-mediated drug–drug interaction with methotrexate. Eur J Pharm Sci.

[37] Fleisher B, Unum J, Shao J, An G (2015). Ingredients in fruit juices interact with dasatinib through inhibition of BCRP: a new mechanism of beverage–drug interaction. J Pharm Sci.

[38] Gozzi GJ, Bouaziz Z, Winter E, Daflon-Yunes N, Aichele D, Nacereddine A, Marminon C, Valdameri G, Zeinyeh W, Bollacke A, Guillon J, Lacoudre A, Pinaud N, Cadena SM, Jose J, Le Borgne M, Di Pietro A (2015). Converting potent indeno 1,2-b indole inhibitors of protein kinase CK2 into selective inhibitors of the breast cancer resistance protein ABCG2. J Med Chem.

[39] Gozzi GJ, Bouaziz Z, Winter E, Daflon-Yunes N, Honorat M, Guragossian N, Marminon C, Valdameri G, Bollacke A, Guillon J, Pinaud N, Marchivie M, Cadena SM, Jose J, Le Borgne M, Di Pietro A (2015). Phenolic indeno 1,2-b indoles as ABCG2-selective potent and non-toxic inhibitors stimulating basal ATPase activity. Drug Des Dev Ther.

[40] Gros G, Martinez L, Gimenez AS, Adler P, Maurin P, Wolkowicz R, Falson P, Hasserodt J (2013). Modular construction of quaternary hemiaminal-based inhibitor candidates and their in cellulo assessment with HIV-1 protease. Bioorg Med Chem.

[41] Gu X, Ren Z, Peng H, Peng S, Zhang Y (2014). Bifendate-chalcone hybrids: a new class of potential dual inhibitors of P-glycoprotein and breast cancer resistance protein. Biochem Biophys Res Commun.

[42] Gu X, Tang X, Zhao Q, Peng H, Peng S, Zhang Y (2014). Discovery of alkoxyl biphenyl derivatives bearing dibenzo c, e azepine scaffold as potential dual inhibitors of P-glycoprotein and breast cancer resistance protein. Bioorg Med Chem Lett.

[43] Gujarati NA, Zeng L, Gupta P, Chen Z-S, Korlipara VL (2017). Design, synthesis and biological evaluation of benzamide and phenyltetrazole derivatives with amide and urea linkers as BCRP inhibitors. Bioorg Med Chem Lett.

[44] Han Y, Riwanto M, Go M-L, Ee PLR (2008). Modulation of breast cancer resistance protein (BCRP/ABCG2) by non-basic chalcone analogues. Eur J Pharm Sci.

[45] Hayashi D, Tsukioka N, Inoue Y, Matsubayashi Y, Iizuka T, Higuchi K, Ikegami Y, Kawasaki T (2015). Synthesis and ABCG2 inhibitory evaluation of 5-*N*-acetylardeemin derivatives. Bioorg Med Chem.

[46] Henrich CJ, Bokesch HR, Dean M, Bates SE, Robey RW, Goncharova EI, Wilson JA, McMahon JB (2006). A high-throughput cell-based assay for inhibitors of ABCG2 activity. J Biomol Screen.

[47] Henrich CJ, Robey RW, Takada K, Bokesch HR, Bates SE, Shukla S, Ambudkar SV, McMahon JB, Gustafson KR (2009). Botryllamides: natural product inhibitors of ABCG2. ACS Chem Biol.

[48] Juvale K, Gallus J, Wiese M (2013). Investigation of quinazolines as inhibitors of breast cancer resistance protein (ABCG2). Bioorg Med Chem.

[49] Juvale K, Stefan K, Wiese M (2013). Synthesis and biological evaluation of flavones and benzoflavones as inhibitors of BCRP/ABCG2. Eur J Med Chem.

[50] Karthikeyan C, Malla R, Ashby CR, Amawi H, Abbott KL, Moore J, Chen J, Balch C, Lee C, Flannery PC, Trivedi P, Faridi JS, Pondugula SR, Tiwari AK (2016). Pyrimido 1″, 2″:1,5 pyrazolo 3,4-b quinolines: novel compounds that reverse ABCG2-mediated resistance in cancer cells. Cancer Lett.

[51] Kee WT, Cooney J, Jensen D, Yan L, Paxton JW, Birch NP, Scheepens A (2014). Hop-derived prenylflavonoids are substrates and inhibitors of the efflux transporter breast cancer resistance protein (BCRP/ABCG2). Mol Nutr Food Res.

[52] Koehler SC, Silbermann K, Wiese M (2016). Phenyltetrazolyl-phenylamides: substituent impact on modulation capability and selectivity toward the efflux protein ABCG2 and investigation of interaction with the transporter. Eur J Med Chem.

[53] Koehler SC, Vandati S, Scholz MS, Wiese M (2018). Structure activity relationships, multidrug resistance reversal and selectivity of heteroarylphenyl ABCG2 inhibitors. Eur J Med Chem.

[54] Koehler SC, Wiese M (2015). HM30181 derivatives as novel potent and selective inhibitors of the breast cancer resistance protein (BCRP/ABCG2). J Med Chem.

[55] Kraege S, Koehler SC, Wiese M (2016). Acryloylphenylcarboxamides: a new class of breast cancer resistance protein (ABCG2) modulators. ChemMedChem.

[56] Kraege S, Stefan K, Juvale K, Ross T, Willmes T, Wiese M (2016). The combination of quinazoline and chalcone moieties leads to novel potent heterodimeric modulators of breast cancer resistance protein (BCRP/ABCG2). Eur J Med Chem.

[57] Kraege S, Stefan K, Koehler SC, Wiese M (2016). Optimization of Acryloylphenylcarboxamides as inhibitors of ABCG2 and comparison with acryloylphenylcarboxylates. ChemMedChem.

[58] Krapf MK, Gallus J, Vahdati S, Wiese M (2018). New inhibitors of breast cancer resistance protein (ABCG2) containing a 2,4-disubstituted pyridopyrimidine scaffold. J Med Chem.

[59] Krapf MK, Gallus J, Wiese M (2017). Synthesis and biological investigation of 2,4-substituted quinazolines as highly potent inhibitors of breast cancer resistance protein (ABCG2). Eur J Med Chem.

[60] Krapf MK, Gallus J, Wiese M (2017). 4-Anilino-2-pyridylquinazolines and -pyrimidines as highly potent and nontoxic inhibitors of breast cancer resistance protein (ABCG2). J Med Chem.

[61] Krapf MK, Wiese M (2016). Synthesis and biological evaluation of 4-anilino-quinazolines and -quinolines as inhibitors of breast cancer resistance protein (ABCG2). J Med Chem.

[62] Krauze A, Grinberga S, Krasnova L, Adlere I, Sokolova E, Domracheva I, Shestakova I, Andzans Z, Duburs G (2014). Thieno 2,3-b pyridines-A new class of multidrug resistance (MDR) modulators. Bioorg Med Chem.

[63] Li X-Q, Wang L, Lei Y, Hu T, Zhang F-L, Cho C-H, To KKW (2015). Reversal of P-gp and BCRP-mediated MDR by tariquidar derivatives. Eur J Med Chem.

[64] Li Y, Woo J, Chmielecki J, Xia CQ, Liao M, Chuang B-C, Yang JJ, Guan MY, Plesescu M, Prakash SR (2016). Synthesis of a new inhibitor of breast cancer resistance protein with significantly improved pharmacokinetic profiles. Bioorg Med Chem Lett.

[65] Marighetti F, Steggemann K, Karbaum M, Wiese M (2015). Scaffold identification of a new class of potent and selective BCRP inhibitors. ChemMedChem.

[66] Miyata H, Takada T, Toyoda Y, Matsuo H, Ichida K, Suzuki H (2016). Identification of febuxostat as a new strong ABCG2 inhibitor: potential applications and risks in clinical situations. Front Pharmacol.

[67] Ochoa-Puentes C, Bauer S, Kuehnle M, Bernhard G, Buschauer A, Koenig B (2013). Benzanilide-biphenyl replacement: a bioisosteric approach to quinoline carboxamide-type ABCG2 modulators. ACS Med Chem Lett.

[68] Pires ARA, Lecerf-Schmidt F, Guragossian N, Pazinato J, Gozzi GJ, Winter E, Valdameri G, Veale A, Boumendjel A, Di Pietro A, Peres B (2016). New, highly potent and non-toxic, chromone inhibitors of the human breast cancer resistance protein ABCG2. Eur J Med Chem.

[69] Revalde JL, Li Y, Hawkins BC, Rosengren RJ, Paxton JW (2015). Heterocyclic cyclohexanone monocarbonyl analogs of curcumin can inhibit the activity of ATP-binding cassette transporters in cancer multidrug resistance. Biochem Pharmacol.

[70] Schexnayder C, Stratford RE (2016). Genistein and glyceollin effects on ABCC2 (MRP2) and ABCG2 (BCRP) in Caco-2 cells. Int J Environ Res Public Health.

[71] Schmitt F, Draut H, Biersack B, Schobert R (2016). Halogenated naphthochalcones and structurally related naphthopyrazolines with antitumor activity. Bioorg Med Chem Lett.

[72] Schmitt SM, Stefan K, Wiese M (2016). Pyrrolopyrimidine derivatives as novel inhibitors of multidrug resistance-associated protein 1 (MRP1, ABCC1). J Med Chem.

[73] Sjostedt N, Holvikari K, Tammela P, Kidron H (2017). Inhibition of breast cancer resistance protein and multidrug resistance associated protein 2 by natural compounds and their derivatives. Mol Pharm.

[74] Song JG, Lee YS, Park J-A, Lee E-H, Lim S-J, Yang SJ, Zhao M, Lee K, Han H-K (2016). Discovery of LW6 as a new potent inhibitor of breast cancer resistance protein. Cancer Chemother Pharmacol.

[75] Spindler A, Stefan K, Wiese M (2016). Synthesis and investigation of tetrahydro-beta-carboline derivatives as inhibitors of the breast cancer resistance protein (ABCG2). J Med Chem.

[76] Suzuki M, Suzuki H, Sugimoto Y, Sugiyama Y (2003). ABCG2 transports sulfated conjugates of steroids and xenobiotics. J Biol Chem.

[77] Tan KW, Killeen DP, Li Y, Paxton JW, Birch NP, Scheepens A (2014). Dietary polyacetylenes of the falcarinol type are inhibitors of breast cancer resistance protein (BCRP/ABCG2). Eur J Pharmacol.

[78] Tan KW, Li Y, Paxton JW, Birch NP, Scheepens A (2013). Identification of novel dietary phytochemicals inhibiting the efflux transporter breast cancer resistance protein (BCRP/ABCG2). Food Chem.

[79] Valdameri G, Gauthier C, Terreux R, Kachadourian R, Day BJ, Winnischofer SMB, Rocha MEM, Frachet V, Ronot X, Di Pietro A, Boumendjel A (2012). Investigation of chalcones as selective inhibitors of the breast cancer resistance protein: critical role of methoxylation in both inhibition potency and cytotoxicity. J Med Chem.

[80] Valdameri G, Genoux-Bastide E, Peres B, Gauthier C, Guitton J, Terreux R, Winnischofer SMB, Rocha MEM, Boumendjel A, Di Pietro A (2012). Substituted chromones as highly potent nontoxic inhibitors, specific for the breast cancer resistance protein. J Med Chem.

[81] Wieczorek A, Blauz A, Zakrzewski J, Rychlik B, Plazuk D (2016). Ferrocenyl 2,5-piperazinediones as tubulin-binding organometallic ABCB(1) and ABCG(2) inhibitors active against MDR cells. ACS Med Chem Lett.

[82] Winter E, Gozzi GJ, Chiaradia-Delatorre LD, Daflon-Yunes N, Terreux R, Gauthier C, Mascarello A, Leal PC, Cadena SM, Yunes RA, Nunes RJ, Creczynski-Pasa TB, Di Pietro A (2014). Quinoxaline-substituted chalcones as new inhibitors of breast cancer resistance protein ABCG2: polyspecificity at B-ring position. Drug Des Dev Ther.

[83] Winter E, Lecerf-Schmidt F, Gozzi G, Peres B, Lightbody M, Gauthier C, Ozvegy-Laczka C, Szakacs G, Sarkadi B, Creczynski-Pasa TB, Boumendjel A, Di Pietro A (2013). Structure–activity relationships of chromone derivatives toward the mechanism of interaction with and inhibition of breast cancer resistance protein ABCG2. J Med Chem.

[84] Winter E, Neuenfeldt PD, Chiaradia-Delatorre LD, Gauthier C, Yunes RA, Nunes RJ, Creczynski-Pasa TB, Di Pietro A (2014). Symmetric Bis-chalcones as a new type of breast cancer resistance protein inhibitors with a mechanism different from that of chromones. J Med Chem.

[85] (2015) MOE molecular simulation package. Chemical Computing Group ULC, Montreal

[86] Chen L, Li Y, Zhao Q, Peng H, Hou T (2011). ADME evaluation in drug discovery. 10. Predictions of P-glycoprotein inhibitors using recursive partitioning and Naive bayesian classification techniques. Mol Pharm.

[87] Wang S, Li Y, Wang J, Chen L, Zhang L, Yu H, Hou T (2012). ADMET evaluation in drug discovery. 12. Development of binary classification models for prediction of hERG potassium channel blockage. Mol Pharm.

[88] Li D, Chen L, Li Y, Tian S, Sun H, Hou T (2014). ADMET evaluation in drug discovery. 13. Development of in silico prediction models for P-glycoprotein substrates. Mol Pharm.

[89] Yap CW (2011). PaDEL-descriptor: an open source software to calculate molecular descriptors and fingerprints. J Comput Chem.

[90] Brooks SP, Morgan BJT (1995). Optimization using simulated annealing. Statistician.

[91] Kuhn M. Feature selection using simulated annealing. https://topepo.github.io/caret/feature-selection-using-simulated-annealing.html. Accessed 16 Apr

[92] Kuhn M. Package ‘caret’. https://cran.r-project.org/web/packages/caret/caret.pdf. Accessed 16 Apr

[93] Xia XY, Maliski EG, Gallant P, Rogers D (2004). Classification of kinase inhibitors using a Bayesian model. J Med Chem.

[94] Mitchell JB (2014). Machine learning methods in chemoinformatics. Wiley Interdiscip Rev Comput Mol Sci.

[95] Ren Y, Zhou L, Yang L, Liu P, Zhao B, Liu H (2016). Predicting the aquatic toxicity mode of action using logistic regression and linear discriminant analysis. SAR QSAR Environ Res.

[96] Friedman JH (2002). Stochastic gradient boosting. Comput Stat Data Anal.

[97] Dahl GE, Jaitly N, Salakhutdinov R. Multi-task neural networks for QSAR predictions. arXiv e-prints. 2014. https://ui.adsabs.harvard.edu/abs/2014arXiv1406.1231D. Accessed 01 June 2014

[98] Snoek J, Larochelle H, Adams RP (2012). Practical bayesian optimization of machine learning algorithms. Adv Neural Inf Process Syst.

[99] Koutsoukas A, Monaghan KJ, Li X, Huan J (2017). Deep-learning: investigating deep neural networks hyper-parameters and comparison of performance to shallow methods for modeling bioactivity data. J Cheminform.

[100] Guidance document on the validation of (quantitative) structure–activity relationship [(Q) SAR] models. OECD Series on Testing and Assessment, 1–154, 2014

[101] Mayr A, Klambauer G, Unterthiner T, Hochreiter S (2016). DeepTox: toxicity prediction using deep learning. Front Environ Sci.

[102] Mayr A, Klambauer G, Unterthiner T, Steijaert M, Wegner JK, Ceulemans H, Clevert D-A, Hochreiter S (2018). Large-scale comparison of machine learning methods for drug target prediction on ChEMBL. Chem Sci.

[103] Jaworska J, Nikolova-Jeliazkova N, Aldenberg T (2005). QSAR applicability domain estimation by projection of the training set in descriptor space: a review. Atla-Altern Lab Anim.

[104] Sahigara F, Mansouri K, Ballabio D, Mauri A, Consonni V, Todeschini RJM (2012). Comparison of different approaches to define the applicability domain of QSAR models. Molecules.

[105] Nina Nikolova-Jeliazkova JJ (2006) AmbitDiscovery-v0.04

[106] Lei T, Sun H, Kang Y, Zhu F, Liu H, Zhou W, Wang Z, Li D, Li Y, Hou T (2017). ADMET evaluation in drug discovery. 18. Reliable prediction of chemical-induced urinary tract toxicity by boosting machine learning-approaches. Mol Pharm.

[107] Fisher A, Rudin C, Dominici F (2018) All models are wrong but many are useful: variable importance for black-box, proprietary, or misspecified prediction models, using model class reliance. arXiv preprint arXiv:1801.01489

[108] Burzykowski PBT. Chapter 15 feature importance. https://pbiecek.github.io/PM_VEE/featureImportance.html. Accessed 24 Sept

[109] Tian S, Wang J, Li Y, Li D, Xu L, Hou T (2015). The application of in silico drug-likeness predictions in pharmaceutical research. Adv Drug Deliv Rev.

[110] Jensen BF, Vind C, Padkjær SB, Brockhoff PB, Refsgaard HH (2007). In silico prediction of cytochrome P450 2D6 and 3A4 inhibition using Gaussian kernel weighted k-nearest neighbor and extended connectivity fingerprints, including structural fragment analysis of inhibitors versus noninhibitors. J Med Chem.

[111] Zhang C, Cheng F, Li W, Liu G, Lee PW, Tang Y (2016). In silico prediction of drug induced liver toxicity using substructure pattern recognition method. Mol Inform.

[112] Hegedus C, Szakacs G, Homolya L, Orban TI, Telbisz A, Jani M, Sarkadi B (2009). Ins and outs of the ABCG2 multidrug transporter: an update on in vitro functional assays. Adv Drug Deliv Rev.

[113] Ding Y-L, Shih Y-H, Tsai F-Y, Leong MK (2014). In silico prediction of inhibition of promiscuous breast cancer resistance protein (BCRP/ABCG2). PLoS ONE.

[114] Wei Y, Ma Y, Zhao Q, Ren Z, Li Y, Hou T, Peng H (2012). New use for an old drug: inhibiting ABCG2 with sorafenib. Mol Cancer Ther.

[115] Matsson P, Pedersen JM, Norinder U, Bergstrom CAS, Artursson P (2009). Identification of novel specific and general inhibitors of the three major human ATP-binding cassette transporters P-gp, BCRP and MRP2 among registered drugs. Pharm Res.

[116] Yoshikawa M, Ikegami Y, Hayasaka S, Ishii K, Ito A, Sano K, Suzuki T, Togawa T, Yoshida H, Soda H (2004). Novel camptothecin analogues that circumvent ABCG2-associated drug resistance in human tumor cells. Int J Cancer.

[117] Wildman SA, Crippen GM (1999). Prediction of physicochemical parameters by atomic contributions. J Chem Inf Comput Sci.

[118] Lenselink EB, ten Dijke N, Bongers B, Papadatos G, van Vlijmen HWT, Kowalczyk W, Ijzerman AP, van Westen GJP (2017). Beyond the hype: deep neural networks outperform established methods using a ChEMBL bioactivity benchmark set. J Cheminform.

[119] Montanari F, Zdrazil B, Digles D, Ecker GF (2016). Selectivity profiling of BCRP versus P-gp inhibition: from automated collection of polypharmacology data to multi-label learning. J Cheminform.

[120] Wu Z, Lei T, Shen C, Wang Z, Cao D, Hou T (2019). ADMET evaluation in drug discovery. 19. Reliable prediction of human cytochrome P450 inhibition using artificial intelligence approaches. J Chem Inf Model.

[121] Sedykh A, Fourches D, Duan J, Hucke O, Garneau M, Zhu H, Bonneau P, Tropsha A (2013). Human intestinal transporter database: QSAR modeling and virtual profiling of drug uptake, efflux and interactions. Pharm Res.

[122] Eric S, Kalinic M, Ilic K, Zloh M (2014). Computational classification models for predicting the interaction of drugs with P-glycoprotein and breast cancer resistance protein. SAR QSAR Environ Res.

[123] Gimadiev TR, Madzhidov TI, Marcou G, Varnek A (2016). Generative topographic mapping approach to modeling and chemical space visualization of human intestinal transporters. BioNanoScience.

