# Supplementary material for: ADMET evaluation in drug discovery. 20. Prediction of breast cancer resistance protein inhibition through machine learning
Source: J Cheminform. 2020 Mar 5;12:16. doi: 10.1186/s13321-020-00421-y (PMC7059329; doi:10.1186/s13321-020-00421-y)
Supplement: Supplementary file 2 — Additional file 2. The R source code that implements the workflow. [file 13321_2020_421_MOESM2_ESM.zip › Source Code/Source Code/README.docx]

The **Source Code.R** includes the following files: **pre_fs.R** was used to conduct feature pre-process on the whole data set and feature selection only on the training set; **dnn_modeling.R** was used to conduct Bayesian optimization of DNN model and finally modeling based on the optimal hyper-parametres, it is similar for **knn_modeling.R** (k-NN), **nb_modeling.R** (NB), **rlr_modeling.R** (RLR), **sgb_modeling.R** (SGB), **svm_modeling.R** (SVM), and **xgb_modeling.R** (XGBoost); **pca_analysis.R** was used to explore the chemical space distributions of the training and test sets based on principal component analysis (PCA) and scattered distributions of molecular weight and SlogP; **model_interpretation.R** was used to explore and analyze the residual distributions and important features of the four well-performing models (SVM, DNN, XGBoost and SGB), and conduct consensus modeling based on the SVM, DNN and XGBoost model.

The **cluster_analysis.ipynb** and **cluster_analysis_R.ipynb** are the jupyter notebooks for cluster cross-validation analysis of seven explored machine learning methods.

All the computing was implemented in the open-source program: R (version 3.5.3 x64) installed the following packages,

"randomForest" "4.6-14"

"doParallel" "1.0.15"

"caret" "6.0-84"

"e1071" "1.7-2"

"dplyr" "0.8.3"

"pROC" "1.15.3"

"h2o" "3.26.0.2"

"mlrMBO" "1.1.2"

"DiceKriging" "1.5.6"

"kknn" "1.3.1"

"naivebayes" "0.9.6"

"LiblineaR" "2.10-8"

"gbm" "2.1.5"

"kernlab" "0.9-27"

"xgboost" "0.90.0.2"

"psych" "1.8.12"

"ggplot2" "3.2.1"

"gridExtra" "2.3"

"DALEX" "0.4.7"

with the exception of **cluster_analysis.ipynb,** where the clustering of compounds was implemented in the scikit-learn package of Python (version 3.6.5 x64) software.
